# Bottom-up evolution of perovskite clusters into high-activity rhodium nanoparticles toward alkaline hydrogen evolution

**DOI:** 10.1038/s41467-023-35783-y

**Published:** 2023-01-17

**Authors:** Gaoxin Lin, Zhuang Zhang, Qiangjian Ju, Tong Wu, Carlo U. Segre, Wei Chen, Hongru Peng, Hui Zhang, Qiunan Liu, Zhi Liu, Yifan Zhang, Shuyi Kong, Yuanlv Mao, Wei Zhao, Kazu Suenaga, Fuqiang Huang, Jiacheng Wang

**Affiliations:** 1 State Key Lab of High Performance Ceramics and Superfine microstructure, Shanghai Institute of Ceramics, Chinese Academy of Sciences, 201899 Shanghai, China; 2 Center of Materials Science and Optoelectronics Engineering, University of Chinese Academy of Sciences, 100049 Beijing, China; 3 Department of Physics & Center for Synchrotron Radiation Research and Instrumentation, Illinois Institute of Technology, Chicago, IL 60616 USA; 4 Department of Mechanical, Materials and Aerospace Engineering, Illinois Institute of Technology, Chicago, IL 60616 USA; 5 School of Physical Science and Technology, ShanghaiTech University, 201210 Shanghai, China; 6 State Key Laboratory of Functional Materials for Informatics, Shanghai Institute of Microsystem and Information Technology, Chinese Academy of Sciences, 200050 Shanghai, China; 7 SANKEN, Osaka University, Ibaraki, 567-0047 Japan; 8 State Key Laboratory of Rare Earth Materials Chemistry and Applications, College of Chemistry and Molecular Engineering, Peking University, 100871 Beijing, China; 9 Hebei Provincial Key Laboratory of Inorganic Nonmetallic Materials, College of Materials Science and Engineering, North China University of Science and Technology, 063210 Tangshan, China; 10 School of Materials Science and Engineering, Taizhou University, 318000 Taizhou, Zhejiang China

**Keywords:** Electrocatalysis, Electrocatalysis, Electrochemistry, Energy

## Abstract

Self-reconstruction has been considered an efficient means to prepare efficient electrocatalysts in various energy transformation process for bond activation and breaking. However, developing nano-sized electrocatalysts through complete in-situ reconstruction with improved activity remains challenging. Herein, we report a bottom-up evolution route of electrochemically reducing Cs_3_Rh_2_I_9_ halide-perovskite clusters on N-doped carbon to prepare ultrafine Rh nanoparticles (~2.2 nm) with large lattice spacings and grain boundaries. Various in-situ and ex-situ characterizations including electrochemical quartz crystal microbalance experiments elucidate the Cs and I extraction and Rh reduction during the electrochemical reduction. These Rh nanoparticles from Cs_3_Rh_2_I_9_ clusters show significantly enhanced mass and area activity toward hydrogen evolution reaction in both alkaline and chlor-alkali electrolyte, superior to liquid-reduced Rh nanoparticles as well as bulk Cs_3_Rh_2_I_9_-derived Rh via top-down electro-reduction transformation. Theoretical calculations demonstrate water activation could be boosted on Cs_3_Rh_2_I_9_ clusters-derived Rh nanoparticles enriched with multiply sites, thus smoothing alkaline hydrogen evolution.

## Abstract (web summary)

Self-reconstruction is an efficient method to synthesize active electrocatalysts. Here, the authors demonstrate a bottom-up evolution route of electrochemically reducing Cs_3_Rh_2_I_9_ halide-perovskite clusters to prepare ultrafine Rh nanoparticles with multiply sites for alkaline hydrogen evolution.

## Introduction

Hydrogen, as an energy carrier, is critical to utilize the renewable energy including wind, solar and hydropower for sustainable development^1–6^. The alkaline hydrogen evolution reaction (HER) with sluggish kinetics limits the efficiency of H_2_ generation in water electrolysis and chlor-alkali electrolysis due to the additional step of water dissociation^7–12^. It starts from the cleaving of the H−OH bond coupled with an electron to form the adsorbed hydrogen atom (H_ad_, Volmer step). Then, H_2_ is produced by the combination of two H_ad_ (Tafel step) or the interaction of H_ad_ and a water molecule (Heyrovsky step)^7,13–15^. Thus, the properties of water dissociation, and hydroxy radical and hydrogen adsorption are crucial for alkaline HER.

To achieve high activity toward these basic steps in alkaline HER, it is necessary to design a functional composite with multiple active centers to facilitate the overall reaction^16–21^. However, such a comprehensive system not only brings difficulty in material synthesis, but also is harmful to the durability of the catalyst due to element or phase segregation^22–26^, especially in a harsh chlor-alkali solution. The potential-driven structural reconstruction of a pre-catalyst during the working conditions is an efficient method to enhance the electrochemical performance^26–29^. The reconstruction of pre-catalysts could obtain the amorphous or defect-rich structure, increased accessible surface area, optimized adsorption properties, and promoted charge transfer^30–33^, thus leading to the improvement of activity and stability. For example, Ni_2_P could be transformed in situ into Ni_2_P/NiO_x_ core-shell structure during oxygen evolution reaction^34^. It could accelerate water adsorption and dissociation kinetics due to unique heterostructures and multiple active centers. Besides, the SrIrO_3_ perovskite electrode experiences self-reconstruction during the alkaline HER because of lattice Sr^2+^ leaching^35^. The formed metallic Ir on the surface of perovskite results in the remarkable activity enhancement as well as excellent stability. However, most pre-catalysts undergo the surface-reconstruction, which leads to the low component utilization as the inert internal part is inaccessible to the surface catalysis. Moreover, the reconstruction degree is highly relevant to the reaction environment, and it may be changed with pH, temperature, electrolyte, and applied potential^31,36–38^, which is disadvantageous to the industrial extreme condition. Therefore, the electrocatalysts consisting of single-component nanoparticles prepared via complete in situ reconstruction are highly desired to avoid the above problems during the alkaline HER.

**Fig. 1:**
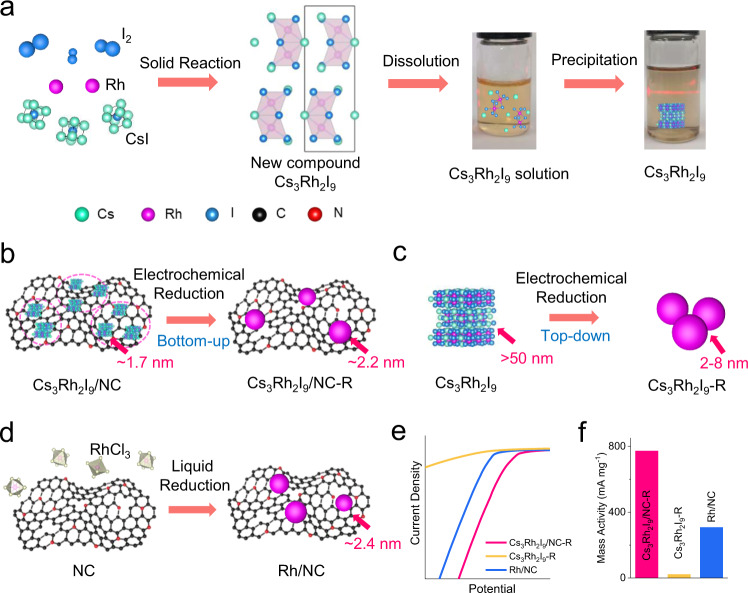
Schematic illustration of synthesizing perovskite Cs_3_Rh_2_I_9_ bulk crystals and clusters Cs_3_Rh_2_I_9_/NC, and their electrochemical reduction into metallic Rh particles toward alkaline HER. **a** Synthesis of Cs_3_Rh_2_I_9_ bulk crystal via a solid reaction using I_2_, Rh, and CsI at 800 ^o^C. The Cs_3_Rh_2_I_9_ crystal could demonstrate a dissolution-precipitation phenomenon in N, N-dimethylformamide (DMF). **b** Electrochemical reduction of Cs_3_Rh_2_I_9_ clusters (~1.7 nm) supported on NC (Cs_3_Rh_2_I_9_/NC) to form Cs_3_Rh_2_I_9_/NC-R with a little larger size (~2.2 nm) via a bottom-up evolution route. **c** Electrochemical reduction of bulk Cs_3_Rh_2_I_9_ to form Cs_3_Rh_2_I_9_-R with vert large particle size via a top-down route. **d** Liquid reduction of RhCl_3_ in aqueous to form Rh/NC with average particle size of 2.4 nm as the control catalyst. **e** HER polarization curves of Cs_3_Rh_2_I_9_/NC-R, Cs_3_Rh_2_I_9_-R, and Rh/NC coated on rotating glassy carbon electrode (GCE, 1600 rpm) in a chlorine-alkali electrolyte. **f** HER mass activity comparison in a chlorine-alkali electrolyte for Cs_3_Rh_2_I_9_/NC-R, Cs_3_Rh_2_I_9_-R, and Rh/NC electrocatalysts at an overpotential of 50 mV.

Herein, we develop a bottom-up evolution route to prepare high-activity Rh nanoparticles via in situ electrochemical reduction of new Cs_3_Rh_2_I_9_ perovskite clusters. These electrochemically reduced Rh nanoparticles could be used as a highly efficient HER catalyst in both alkaline and chlor-alkali electrolyte. The new halide-perovskite compound Cs_3_Rh_2_I_9_ with dimer unit [Rh_2_I_9_]^3−^ separated by Cs ions as the pre-catalyst of electrochemically synthesized Rh nanoparticles was synthesized by solid state reaction (Fig. 1a). It could be dissolved in N, N-dimethylformamide (DMF). The unique zero-dimensional structure allows it to be downsized into small clusters on a polar nitrogen-doped carbon (NC) support by a simple dissolution-precipitation method (Fig. 1b). The high surface energy of Cs_3_Rh_2_I_9_ clusters on NC (Cs_3_Rh_2_I_9_/NC) could promote an electrochemical self-reduction and bottom-up evolution, leading to the formation of unique Rh nanoparticles with larger lattice spacings and lower atomic coordination number (Fig. 1b). In sharp contrast, such Rh nanoparticles cannot be formed from bulk Cs_3_Rh_2_I_9_ and liquid reduction of RhCl_3_ by NaBH_4_ (Fig. 1c, d). The complete reconstructed Cs_3_Rh_2_I_9_/NC-R could significantly reduce the barrier of water dissociation in alkaline HER. Therefore, Cs_3_Rh_2_I_9_/NC-R exhibits high mass activity of 772.1 mA mg^−1^_Rh_ in a chlorine-alkali electrolyte, which is about 2.5 times and 35.5 times that of liquid-reduced Rh/NC with similar particle size and electrochemically reduced Cs_3_Rh_2_I_9_-R with the larger size, respectively (Fig. 1e, f). And it also shows the negligible activity loss after 50 h durable measurement.

## Results

### Synthesis and characterization of bulk and cluster Cs_3_Rh_2_I_9_

**Fig. 2:**
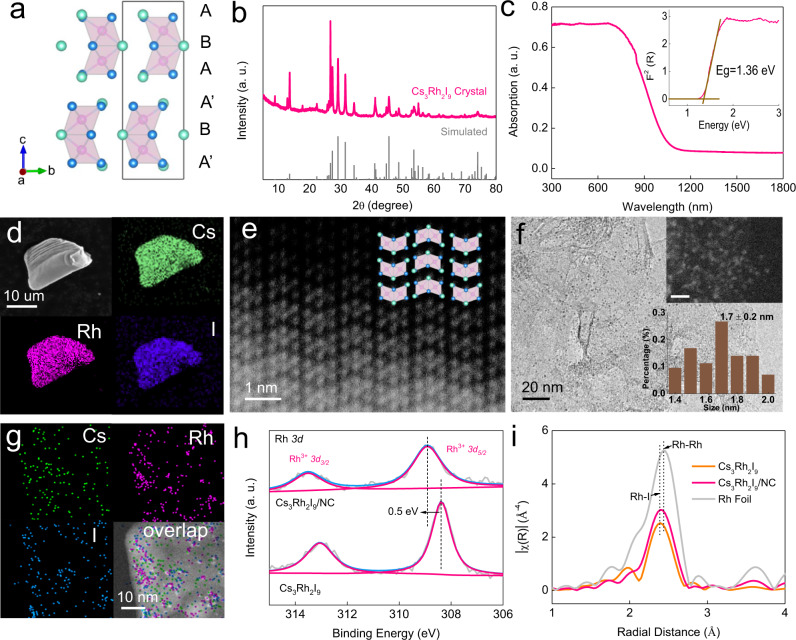
Structure characterization of bulk Cs_3_Rh_2_I_9_ and nanoclusters Cs_3_Rh_2_I_9_/NC. **a** Atomic structure, and (**b**) powder XRD pattern and simulated pattern of Cs_3_Rh_2_I_9_ crystal. **c** Diffuse-reflectance UV–Vis spectrum of Cs_3_Rh_2_I_9_. The inset shows the corresponding Tauc plot. **d** SEM-EDX mapping images of Cs_3_Rh_2_I_9_ single crystal. **e** HAADF-STEM image of Cs_3_Rh_2_I_9_ crystal. **f** TEM image of Cs_3_Rh_2_I_9_ clusters supported on NC (Cs_3_Rh_2_I_9_/NC, Rh content: 5.8 wt.%). The insets show the corresponding HAADF image (scale bar: 5 nm, up) and particle size distribution (down). **g** TEM-EDX mapping of Cs_3_Rh_2_I_9_/NC. **h** XPS spectra of Rh *3d* for Cs_3_Rh_2_I_9_ and Cs_3_Rh_2_I_9_/NC. **i** Fourier transform EXAFS of Rh K-edge in R-space for Cs_3_Rh_2_I_9_, Cs_3_Rh_2_I_9_/NC, and Rh foil.

As a new compound, Cs_3_Rh_2_I_9_ single crystals were synthesized by the solid state reaction using CsI as the flux. Its structure was determined by single-crystal X-ray diffraction (XRD). It belongs to the hexagonal *P*6_3_*/mmc* space group with *a* = *b* = 7.9648 (14) Å, *c* = 20.028 (4) Å, *V* = 1100.3 (4) Å^3^, *Z* = 2, and a calculated density of *d* = 5.272 g cm^−3^. Details of the atomic coordinates in the compound are shown in Supplementary Table 1, 2. Cs_3_Rh_2_I_9_ with a zero-dimensional structure consists of alternating hexagonal CsI_3_ layers in ABAA’BA’ stacking sequence (Fig. 2a). Two adjacent [RhI_6_]^3−^ octahedrons share three I atoms on the plane B to form a [Rh_2_I_9_]^3−^ dimer. The powder XRD pattern of Cs_3_Rh_2_I_9_ is consistent with the simulated result (Fig. 2b), confirming its zero-dimensional perovskite structure^39^. Cs_3_Rh_2_I_9_ shows a semiconductor behavior with an optical band gap of 1.36 V (Fig. 2c)^40^. Single-crystal Cs_3_Rh_2_I_9_ is diamagnetic due to the d^6^ electronic configuration of Rh^3+^ (Supplementary Fig. 2). Energy dispersive X-ray (EDX) spectroscopy shows homogenous distribution of Cs, Rh, and I elements in crystalline Cs_3_Rh_2_I_9_ (Fig. 2d) and EDX results from TEM show Cs, Rh and I with an atomic ratio of ~3:2:9, consistent with the stoichiometry of Cs_3_Rh_2_I_9_ (Supplementary Fig. 3 and Table 3). The high-angle annular dark-field scanning transmission electron microscopy (HAADF-STEM) confirms the alternating CsI_3_ layer structure (Fig. 2e). Cs_3_Rh_2_I_9_ also exhibits good stability as its structure does not change in 1.0 M HCl or KOH for 7 days (Supplementary Fig. 5).

Cs_3_Rh_2_I_9_ can be dissolved in N, N-dimethylformamide (DMF) to form a brownish yellow solution due to the polar aprotic property of DMF (Supplementary Fig. 6). When adding water, Cs_3_Rh_2_I_9_ precipitates without any change of structure (Supplementary Fig. 7, 8 and 10). By this means, the use of polar nitrogen-doped carbon (NC) during the precipitation process can significantly reduce the particle size to form Cs_3_Rh_2_I_9_ nanoclusters on NC (Cs_3_Rh_2_I_9_/NC). Figure 2f shows the uniform distribution of Cs_3_Rh_2_I_9_ nanoclusters with a size of ~1.7 nm. No obvious particles were observed in SEM images, further confirming the uniform and ultra-small size of Cs_3_Rh_2_I_9_ (Supplementary Fig. 9). The structure and composition of Cs_3_Rh_2_I_9_/NC is the same as Cs_3_Rh_2_I_9_ (Fig. 2g and Supplementary Fig. 10). The X-ray photoelectron spectroscopy (XPS) spectra show a positive core level shift of about 0.5 eV for Cs_3_Rh_2_I_9_/NC (Fig. 2h), indicating the strong carrier effect of Cs_3_Rh_2_I_9_ clusters on the NC^41^. The Rh−I coordination was identified by extended X-ray absorption fine structure (EXAFS; Fig. 2i). The shell at 2.40 Å represents the Rh−I scattering path for Cs_3_Rh_2_I_9_ and Cs_3_Rh_2_I_9_/NC, while the fitting results suggest that an additional Rh−Rh scattering path appears in Cs_3_Rh_2_I_9_/NC (Supplementary Fig. 13 and Table 4). This may be caused by partial decomposition of Cs_3_Rh_2_I_9_ nanoclusters under the high-energy measurement (Supplementary Fig. 14).

### Electrochemical reduction of Cs_3_Rh_2_I_9_

**Fig. 3:**
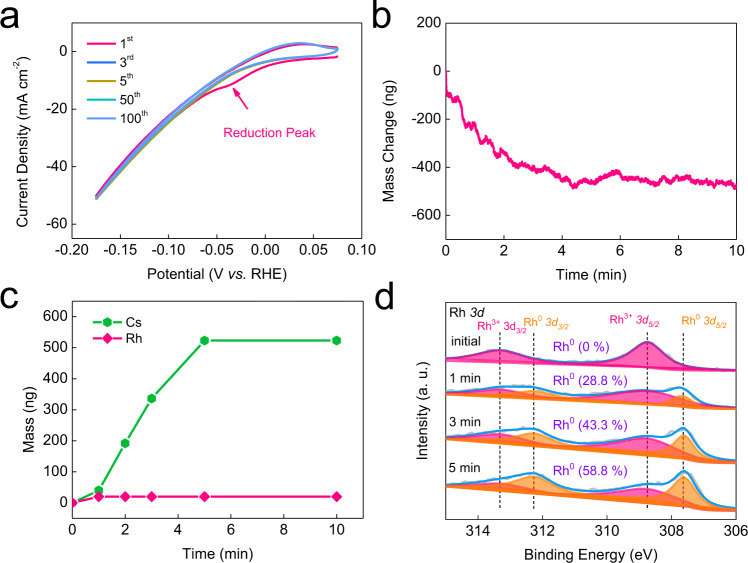
Reconstruction of clusters Cs_3_Rh_2_I_9_/NC into Rh nanoparticles on NC. **a** The CV curves from 1^st^ to 100^th^ cycle at 100 mV s^−1^ for Cs_3_Rh_2_I_9_/NC in 1.0 M KOH. **b** Mass change of the Cs_3_Rh_2_I_9_/NC electrode monitored by in situ EQCM experiment. **c** ICP-MS of the Cs and Rh contents in the electrolyte at different reduction time. **d** XPS spectra of Rh *3d* for Cs_3_Rh_2_I_9_/NC at different reduction time. The results in (**b**–**d**) were obtained at the potentiostatic measurement at −0.03 V vs. RHE.

Under cathodic potentials, it was found that both Cs_3_Rh_2_I_9_ clusters and single crystals are unstable and could be transformed into Rh particles. Especially, the Cs_3_Rh_2_I_9_ nanoclusters on NC can be electrochemically reduced and assembled into Rh nanoparticles with mean particle size of 2.2 nm via a bottom-up evolution route (Fig. 1b). The reduction peak at about −0.035 V versus reversible hydrogen electrode (vs. RHE) in the first CV curve corresponds to the reduction of Rh^3+^ (Fig. 3a). The in situ Raman experiments indicate that the characteristic structure of Cs_3_Rh_2_I_9_ disappears when the negative potential was applied (Supplementary Fig. 15). To elucidate the process of reconstruction, the reduction was conducted by the potentiostatic measurement at −0.03 V vs. RHE. The in situ electrochemical Quartz Crystal Microbalance (EQCM) experiment indicates that the quality of the electrode continuously decreases and then stabilizes after about 5 min (Fig. 3b). The Inductively Coupled Plasma Mass Spectrometry (ICP-MS) results show the content of Cs^+^ ions in the electrolyte continuously increases in the initial 5 min while Rh in the electrolyte was barely detected (Fig. 3c). And the XPS spectra shows neither Cs nor I was detected after reduction (Supplementary Fig. 16), which is also confirmed by ICP-MS results (Supplementary Table 5). Rh mass content in Cs_3_Rh_2_I_9_/NC-R was determined by the ICP-MS to be 5.7 ± 0.8 wt.%, which is very close to the initial loading of 5.8 wt.%. This indicates all Cs_3_Rh_2_I_9_ nanoclusters could be electrochemically reduced to metallic Rh^0^ nanoparticles. And such a reconstruction process is complete. The ex-situ XPS spectra verify the content of Rh^3+^ decreases while the content of Rh^0^ increases with the reduction time (Fig. 3d and Supplementary Fig. 17).

**Fig. 4:**
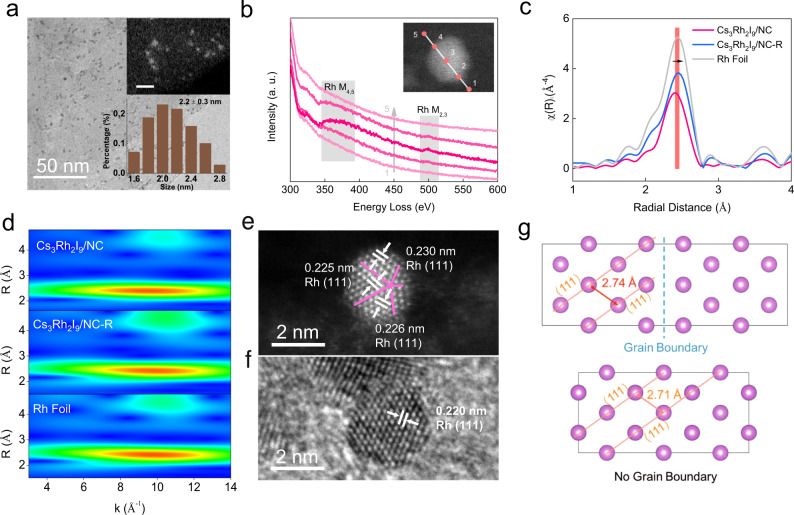
Characterization of Cs_3_Rh_2_I_9_/NC-R prepared by electrochemical reduction of Cs_3_Rh_2_I_9_/NC. **a** TEM image of Cs_3_Rh_2_I_9_/NC-R, showing uniform dispersion of Rh nanoparticles. The inset shows the corresponding HAADF image (scale bar: 5 nm, up) and particle size distribution (down). **b** The linear electron energy loss spectroscopy of Cs_3_Rh_2_I_9_/NC-R. **c** Fourier transform EXAFS of the Rh K-edge in R-space for Cs_3_Rh_2_I_9_/NC, Cs_3_Rh_2_I_9_/NC-R and Rh foil. **d** WT-EXAFS images of Rh for Cs_3_Rh_2_I_9_/NC, Cs_3_Rh_2_I_9_/NC-R and Rh foil. **e** HAADF-STEM image of Cs_3_Rh_2_I_9_/NC-R, showing a single Rh nanoparticle with larger lattice spacing and twin-GBs. **f** HRTEM image of Rh nanoparticles (Cs_3_Rh_2_I_9_-R) derived from the top-down electrochemical reduction of bulk Cs_3_Rh_2_I_9_ crystal. **g** DFT models of Rh with larger lattice spacing and GBs (up panel) and Rh with regular lattice spacing (down panel).

TEM measurements confirm the uniform distribution of Rh particles on the NC after electrochemical reduction (Cs_3_Rh_2_I_9_/NC-R, Fig. 4a and Supplementary Fig. 18) and the Rh particle size (~2.2 nm) is evidently larger than that of Cs_3_Rh_2_I_9_ nanoclusters (~1.7 nm), implying a bottom-up evolution route under cathodic potential. The linear electron energy loss spectroscopy analysis suggests the element in the particle is Rh (Fig. 4b). The bulk Cs_3_Rh_2_I_9_ can also be reconstructed in this way, but it does not reach a stable state within 300 CV cycles due its large size (Supplementary Fig. 19). After reduction (Cs_3_Rh_2_I_9_-R), its edge consists of numerous Rh particles with larger size (4.3 ± 1.2 nm) compared to the product from Cs_3_Rh_2_I_9_/NC (Supplementary Fig. 20, 21). The Rh K-edge X-ray absorption near edge structure spectra indicates the adsorption edge of the reduced Cs_3_Rh_2_I_9_/NC (Cs_3_Rh_2_I_9_/NC-R) shifts to lower energy compared to the initial state (Supplementary Fig. 22). Meanwhile, the shell in EXAFS for the Cs_3_Rh_2_I_9_/NC-R shifts to 2.45 Å (Fig. 4c), similar to that of Rh foil. The fitting results show that its Rh−Rh coordination number is only 8.0 (Fig. 4c and Supplementary Table 6), showing the small particle size. The wavelet transform (WT)-EXAFS analysis shows the first shell of Cs_3_Rh_2_I_9_/NC-R domain at *R* = 2.45 Å and *k* = 9.90 Å^−1^ (Fig. 4d), similar to the Rh foil (*R* = 2.43 Å and *k* = 9.80 Å^−1^) and different from Cs_3_Rh_2_I_9_/NC with Rh−Rh scattering path (*R* = 2.40 Å and *k* = 9.50 Å^−1^).

Moreover, the HAADF-STEM images indicate the Rh nanoparticles in Cs_3_Rh_2_I_9_/NC-R are rich with grain boundaries (GBs) and large lattice spacings (0.225–0.230 nm) (Fig. 4e and Supplementary Fig. 24). In sharp contrast, the particles in electrochemically reduced Cs_3_Rh_2_I_9_ (Cs_3_Rh_2_I_9_-R) form bulk Cs_3_Rh_2_I_9_ show smaller plane spacings of 0.220 nm and no evident GBs could be observed (Fig. 4f and Supplementary Fig. 21). The formation of twinned Rh with large lattice spacings in Cs_3_Rh_2_I_9_/NC-R may be ascribed to the coupling of smaller Rh clusters with high surface energy during the electrochemical reduction process. Limited by the size of the Cs_3_Rh_2_I_9_ cluster (~1.7 nm), the formed Rh clusters could combine with neighboring clusters into particles (~2.2 nm) to decrease the surface energy (Supplementary Fig. 25). However, the Rh particles formed from bulk Cs_3_Rh_2_I_9_ undergo a top-down process of bulk decomposition. They have particle sizes greater than 2 nm and thus are energetically stable enough to not combine into twin crystals. Furthermore, the twinned Rh in Cs_3_Rh_2_I_9_/NC-R exhibits tensile stress with the enlarged lattice fringe of 0.5–5.5% (Fig. 4e, Supplementary Fig. 24). The corresponding GB was established by density functional theory (DFT) calculation (Fig. 4g) to promote tensile stress of the nearby Rh atoms (from 2.71 to 2.74 Å), suggesting the rich GBs in twinned Rh can stabilize the enlarged (111) lattice. The Rh particle with such tensile stress is considered to facilitate H_2_O dissociation in alkaline HER^2^.

### HER activity evaluation

**Fig. 5:**
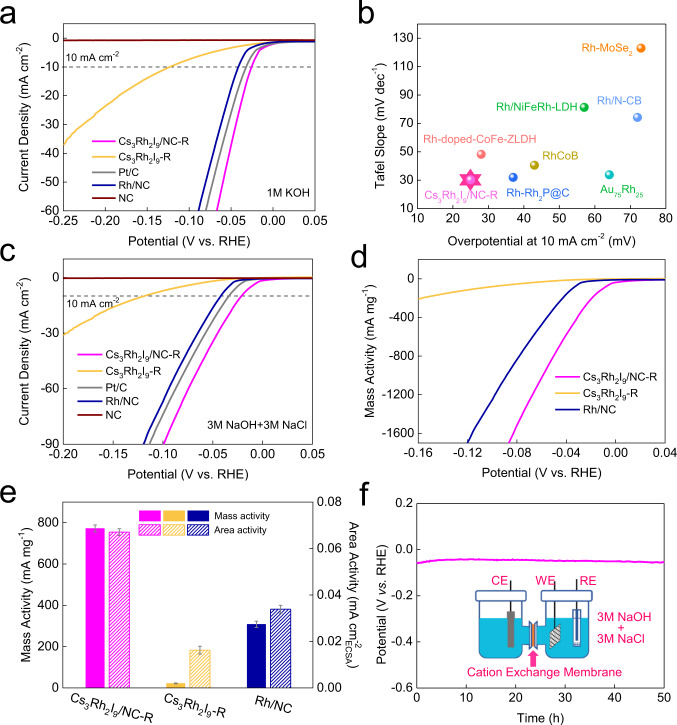
HER activity in 1.0 M KOH and chlor-alkali electrolyte. **a** LSV curves of various electrocatalysts including commercial Pt/C (Pt content: 20 wt.%) coated on rotating glassy carbon electrode (GCE, 1600 rpm) in 1.0 M KOH. The catalyst loading amount on GCE is 0.764 mg cm^−2^. For Rh-based samples, the calculated Rh loading amounts on GCE are 0.045, 0.090 and 0.053 mg cm^−2^ for Cs_3_Rh_2_I_9_/NC-R, Cs_3_Rh_2_I_9_-R, and Rh/NC, respectively. **b** Comparison of overpotential at 10 mA cm^−2^ and Tafel slope for various Rh-based catalysts in 1.0 M KOH. **c** LSV curves of various electrocatalysts coated on rotating GCE (1600 rpm) in chlorine-alkali electrolyte. **d** Mass activity normalized to the mass of Rh in chlorine-alkali electrolyte. **e** Comparison of mass activity and area activity at the overpotential of 50 mV in chlorine-alkali electrolyte. All data show the mean and standard deviation through three repeated measurements. **f** Stability of Cs_3_Rh_2_I_9_/NC-R at 10 mA cm^−2^ in a chlorine-alkali electrolyte.

The HER activity was first evaluated in 1.0 M KOH electrolyte. Before measurement, the electrode was in situ reduced under the CV between 0.075 and -0.175 vs. RHE at a scan rate of 100 mV s^−1^ to achieve a stable stage. The Rh/NC with mean size of ~2.4 nm was synthesized by liquid chemical reduction for comparison (Supplementary Fig. 26). The Rh mass content in Rh/NC is 7 wt.%, and the HRTEM implies the lattice spacing of Rh particle is 0.220 nm, similar to that of Cs_3_Rh_2_I_9_-R without tensile stress. As shown in Fig. 5a, the overpotential at 10 mA cm^−2^ for Cs_3_Rh_2_I_9_/NC-R composed of Rh twin nanoparticles is only 25 mV, evidently lower than those of Cs_3_Rh_2_I_9_-R (123 mV), Pt/C (32 mV) and Rh/NC (41 mV). Moreover, the Tafel slope (30.3 mV dec^−1^) of Cs_3_Rh_2_I_9_/NC-R is also smaller than that of chemically reduced Rh/NC without GBs and tensile stress (34.8 mV dec^−1^) (Supplementary Fig. 28a). It implies that the GBs and tensile stress in Rh nanoparticles have a significant effect on boosting alkaline HER. Besides, Cs_3_Rh_2_I_9_/NC-R exhibits high mass activity of 839.8 mA mg^−1^_Rh_, 21.6 times that of Cs_3_Rh_2_I_9_-R and 2.4 times that of Rh/NC (Supplementary Fig. 28b). The mass loading of Cs_3_Rh_2_I_9_/NCs was also optimized and compared (Supplementary Fig. 29). The electrochemical surface area (ECSA) is enhanced with the increased mass loading, but the optimal activity is achieved at a Rh mass loading of 5.8 wt.% (Cs_3_Rh_2_I_9_/NC-R; Supplementary Fig. 30-32). In addition, the H_2_ production faradaic efficiency for twinned Rh (Cs_3_Rh_2_I_9_/NC-R) was confirmed to be nearly 100% through a drainage method (Supplementary Fig. 33). The HER activity of Cs_3_Rh_2_I_9_/NC-R outperforms most of reported Rh-based electrocatalysts (Fig. 5b) and other advanced alkaline electrocatalysts (Supplementary Table 7).

The HER activity in the simulated chlorine-alkali electrolyte (3.0 M NaOH + 3.0 M NaCl) was further evaluated in a three-electrode system. As shown in Fig. 5c, the overpotentials for Cs_3_Rh_2_I_9_/NC-R are 21, 65, and 107 mV to reach current densities of 10, 50, and 100 mA cm^−2^, respectively, significantly lower than those of Cs_3_Rh_2_I_9_-R, Pt/C, and Rh/NC. The mass activity of Cs_3_Rh_2_I_9_/NC-R is 772.1 mA mg^−1^_Rh_ at −50 mV vs. RHE, outperforming Cs_3_Rh_2_I_9_-R (21.7 mA mg^−1^_Rh_) and Rh/NC (307.8 mA mg^−1^_Rh_) (Fig. 5d, e). The area activity normalized by ECSA of Cs_3_Rh_2_I_9_/NC-R is 0.067 mA cm^−2^, manifesting a factor of 2.0 increase than that of Rh/NC (Fig. 5e and Supplementary Fig. 35b). These results confirm the excellent intrinsic activity of Cs_3_Rh_2_I_9_/NC-R enriched with defects. The enhanced activity may be ascribed to the reconstruction process. Cs_3_Rh_2_I_9_/NC-R with unsaturated atom coordination and more accessible area could accelerate water adsorption and dissociation processes, thus leading to the better performance. Furthermore, Cs_3_Rh_2_I_9_/NC-R exhibits good durability with negligible activity loss after 50 h (Fig. 5f). After the durability measurement, the twin Rh nanoparticles show little agglomeration while the chemical state remains unchanged (Supplementary Fig. 37, 38).

To further disclose the origin of high activity of Cs_3_Rh_2_I_9_/NC-R in the alkaline HER process, the DFT calculation was performed. Based on the above characterizations, the Cs_3_Rh_2_I_9_ clusters-derived Rh nanoparticles possess larger lattice spacings and more GBs that liquid-reduced Rh nanoparticles and Rh particles from electro-reduction of bulk Cs_3_Rh_2_I_9_. Thus, different Rh models with larger lattice spacings and more GBs were built to study the HER mechanism in alkaline. As shown in Supplementary Fig. 39, the increase of H−O−H angle and water molecule binding energy agrees well with the degree of distortion and crystal tension, indicating the enhanced ability of water dissociation. Moreover, the largest H−O−H angle of 105.25˚ is found when water molecule is adsorbed on GBs with low coordination number, which further shows GBs would activate the H−OH bond. The water disassociation, as a pre-reaction to form an adsorbed proton, plays a more critical role in alkaline hydrogen evolution^8,42^. The linear Brønsted–Evans–Polanyi relationship between adsorption energy and dissociative kinetic barrier of H_2_O allows the use of binding energy of water molecule as the activity descriptor for alkaline HER. The sites at GBs and tensed Rh atoms show high H_2_O binding energy. The enhanced water dissociation ability in Cs_3_Rh_2_I_9_/NC-R was further verified by in situ Raman spectra. Supplementary Fig. 40a shows obvious interfacial water on Cs_3_Rh_2_I_9_/NC-R surface in 1 M KOH solution over the potential range from 0 to −0.3 V (vs. RHE). The peaks at 1588 and 1632 cm^−1^ correspond to the G band of carbon substrate and the adsorbed water on Rh, respectively^43,44^. As the potential decreased, the intensity of H–O–H bending increases sharply. However, such a phenomenon is absent in Rh/NC synthesized by chemical reduction (Supplementary Fig. 40b). Thus, the abundant and multiply active sites on twin crystal Rh are effective for cleaving the H−OH bond, contributing to the high electrochemical performance toward HER under alkaline condition.

## Discussion

The defects-rich Rh nanoparticles with average size of ~2.2 nm were prepared by in situ electrochemical reduction of perovskite Cs_3_Rh_2_I_9_ cluster via a bottom-up evolution route. The reduction was investigated by in situ EQCM, ex-situ ICP-MS, and XPS. The as-formed twin crystal Rh nanoparticles with tensile stress exhibits excellent activity and stability in alkaline hydrogen evolution reaction. In 1.0 M KOH, the Cs_3_Rh_2_I_9_/NC-R catalyst showed a low overpotential of 25 mV at the current density of 10 mA cm^−2^ and a small Tafel slope of 30.3 mV dec^−1^. Cs_3_Rh_2_I_9_/NC-R exhibits high mass activity of 839.8 mA mg^−1^_Rh_, 21.6 times that of Cs_3_Rh_2_I_9_-R with bigger size and 2.4 times that of liquid-reduced Rh/NC. In a chlor-alkali electrolyte, the area activity of Cs_3_Rh_2_I_9_/NC-R (0.067 mA cm^−2^_ECSA_ at −50 mV vs. RHE) manifests a factor of 4.1 and 2.0 activity increase compared to Cs_3_Rh_2_I_9_-R and Rh/NC, respectively. Moreover, it exhibits good durability with negligible activity loss for 50 h HER measurement. The DFT calculation revealed that Rh nanoparticles of Cs_3_Rh_2_I_9_/NC-R enriched with multiply catalytic sites could accelerate the activation of adsorbed water molecule, thereby smoothing the whole alkaline HER. The study presents new insights into preparing small-sized nanoparticles via in situ electrochemical reconstruction for energy electrocatalysis.

## Methods

### Synthesis of Cs_3_Rh_2_I_9_ crystal

The Cs_3_Rh_2_I_9_ crystal was prepared *via* solid state reaction. 50 mg rhodium, 185 mg iodine and 1.5 g CsI were mixed by grinding in Ar glovebox to prevent the influence of water. CsI was served as both raw material and flux. The above powder was annealed at 800 °C for 2000 min in the evacuated quartz tube and cooled to room temperature at the rate of 3 °C min^−1^. Finally, the product was washed with deionized water for several times and dried in vacuum at room temperature.

### Synthesis of NC

NC was prepared by the sol–gel method according to our previous report.^41^ It started from mixing 1.8 mL formaldehyde and 1 g melamine in 20 mL deionized water under stirring at 50 °C for 1 h. Then, 4.5 g MnCl_2_ and 6 g PEG were added into the above solution and continuously stirred at room temperature to form a uniform sol. The sol was transferred into a culture dish and dried at 80 °C for 24 h to form a gel precursor. The gel was cut into small slices and pre-carbonized at 400 °C for 2 h in an Ar atmosphere. After grounding the precursor into powder, it was annealed at 900 °C with a heat rate of 3 °C min^−1^ in the Ar atmosphere. The final product NC was obtained by acid treatment to remove the metal impurities.

### Synthesis of nanoclusters Cs_3_Rh_2_I_9_ supported on NC (Cs_3_Rh_2_I_9_/NC)

10 mg Cs_3_Rh_2_I_9_ crystal was added into 40 ml *N*, *N*-dimethylformamide (DMF) and stirred at 60 °C to form the brown solution. Then, 10 mg NC was added and the mixture was continually stirred for 1 h. Subsequently, the above mixture was slowly added into 200 ml H_2_O under fiercely stirring. The induced polar protic solvent with slow S_N_2 reaction kinetics results in the precipitation of Cs_3_Rh_2_I_9_^45^. The Cs_3_Rh_2_I_9_/NC was collected by suction filtration and washed by water and ethanol for several times. And it was dried in vacuum at 60 °C for 12 h. Cs_3_Rh_2_I_9_/NC with different Rh contents (*x* wt.%, *x* = 2.7, 4.3, 5.8, or 7.1) was prepared by changing mass loading of Cs_3_Rh_2_I_9_, and x corresponds to the Rh content. The optimized Cs_3_Rh_2_I_9_/NC (5.8 wt.%) is named as Cs_3_Rh_2_I_9_/NC in the main text and SI if no specific note was mentioned. The re-precipitated Cs_3_Rh_2_I_9_ without adding NC was obtained as the control sample.

### Synthesis of Rh/NC by liquid reduction

RhCl_3_ (7.1 mg) was dissolved in 20 mL N-methyl pyrrolidone (NMP), followed by the addition of 46.5 mg NC. After stirring for 1 h, 0.05 g sodium borohydride in NMP was added into the above solution drop by drop. After stirring for 20 h, Rh/NC was collected by suction filtration and then annealed in 10% H_2_/Ar at 300 °C for 30 min. The elemental analysis shows the mass content of Rh in Rh/NC is 7.0 wt.%.

### Characterization

Single-crystal XRD data was obtained on a Bruker D8 QUEST diffractometer with Mo-*K*_*α*_ (λ = 0.71073 Å) radiation at 300 K. The crystal structure was solved and refined using APEX3 program. Powder XRD was performed on a Bruker D8 Advance diffractometer equipped with mirror-monochromatized source of Cu *Kα* radiation (λ = 0.15406 nm). The ultraviolet–visible (UV–Vis) light diffuse-reflectance spectra were measured on a UV-4100 spectrophotometer operating from 2000 to 300 nm at room temperature and the BaSO_4_ powder was used as a 100% reflectance standard. Low-temperature electrical resistivity was measured using a Physical Properties Measurements System (PPMS-Dyna Cool, Quantum Design). SEM was conducted on the JSM-7800F. TEM was conducted on the JEM-2100F and Talos F200X G2. HAADF-STEM and EELS measurements were obtained from aberration-corrected TEM (Hitachi HF5000) and JEOL Triple-C TEM. The chemical states were investigated by X-ray photoelectron spectroscopy (XPS, Thermo Fisher Scientific ESCA Lab 250Xi spectrometer) with focused monochromatic Al Kα radiation (1486.6 eV, 150 W; 500 μm diameter of irradiated area). Ion concentration in electrolyte was determined by the inductively coupled plasma mass spectrometry (XII, Thermo Fisher Scientific). X-ray absorption fine-structure spectroscopy (XAFS) was performed at the Materials Research Collaborative Access Team (MRCAT), Sector 10-BM line at the Advanced Photon Source at Argonne National Laboratory^46^. Data were processed and fitted using Athena and Artemis for the IFEFFIT suite^47,48^. All spectra were prepared for Fourier Transform using a Hanning window ranging from 2.0 Å^−2^ < k < 14 Å^−1^ with d*k* = 2 Å^−1^ and simultaneously fitted in *k*, *k*^2^, and *k*^3^ weightings using a Hanning window of 1.8 Å < *R* < 2.8 Å with d*R* = 0.2 Å. Wavelet transforms were obtained from processed data using Larch^49^.

### Hydrogen evolution reaction experiments

The electrochemical performance was conducted on the three-electrode system using graphite rod as counter electrode and Hg/HgO as the reference electrode at 25 ^o^C. 5 mg catalyst and 25 uL Nafion (5 wt.%) were added into 475 uL ethanol to form the homogeneous catalyst ink. And the working electrode was prepared by adding 15 uL ink on a rotating GCE (area: 0.1963 cm^−2^). The catalyst loading amount on GCE is 0.764 mg cm^−2^. And the calculated Rh loading amounts on GCE are 0.045, 0.090, and 0.053 mg cm^−2^ for Cs_3_Rh_2_I_9_/NC-R, Cs_3_Rh_2_I_9_-R, and Rh/NC, respectively. Moreover, the commercial Pt/C (Pt content: 20 wt.%) was also used as the control electrocatalyst. The HER measurements were performed in the Ar-saturated electrolyte at the rotating speed of 1600 rpm. Before measurement, the electrode was activated (reduced) under the CV between 0.075 and -0.175 vs. RHE at a scan rate of 100 mV s^−1^ for 100 cycles. The linear sweep voltammetry (LSV) curves were recorded at the scan rate of 5 mV s^−1^ with the IR-compensation 90%. The ECSA was obtained from the equation ECSA = *C*_dl_/*C*_s_, where the electrochemical double layer capacitance (*C*_dl_) was obtained from the CV measurement at different scan rates and the specific capacitance (*C*_s_) was 0.4 μF cm^−2^ in 1.0 M KOH. In chlorine-alkali electrolyte (3.0 M NaOH + 3.0 M NaCl), the Hg/HgO electrode was protected by the salt bridge (1.0 M KOH).

The durable measurement was conducted in a two-compartment cell using the cation exchange membrane as the separator. And the working electrode was prepared by dropping the ink on the carbon cloth (catalyst loading amount: 1 mg cm^−2^).

### Reversible hydrogen electrode (RHE) calibration

The calibration was performed in the H_2_-saturated electrolyte using Pt foils as counter electrode and working electrode. CV measurements were carried out at the scan rate of 1 mV s^−1^. The average potentials at the current of zero were set as the thermodynamic potential of RHE.

### Electrochemical quartz crystal microbalance experiment

The EQCM experiment was conducted on the QSense Explorer instrument (Biolin Scientific AB, Sweden). The working electrode was prepared by spin-coating the catalyst ink on the Au-coated quartz crystal disk (5 MHz). In situ EQCM was measured simultaneously with potentiostatic measurement at −0.03 V vs. RHE.

### Hydrogen production faradic efficiency

The faradic efficiency was determined by the drainage method at 25 °C. A constant current was applied on the electrode, and the electrocatalysis time and the volume of evolved hydrogen were recorded synchronously. Each experiment was repeated three times. The experimental content of produced hydrogen was calculated by the Ideal Gas Law and the theoretical content was determined by the Faraday Law.

### Computational methods

All theoretical calculations were performed using DFT, as is implemented in the Vienna ab initio simulation package (VASP). The electron exchange and correlation energy functionals are treated using the generalized gradient approximation, as is captured using the Perdew-Burke-Ernzerh functional (GGA-PBE). Iterative solutions of the Kohn–Sham equations were done using a plane-wave basis set defined using a kinetic energy cutoff of 500 eV. The k-point sampling was obtained from the Monkhorst–Pack scheme with a (6 × 6 × 1) mesh. respectively. Rh (001) surface structures with lattice tensile of 1.5% and 3% were built and optimized to simulate active sites with different crystal strain. Surface structures with Rh (110) GB and standard Rh (001) were chosen to reflect the properties of GB and monocrystal Rh, respectively. Water bonding energy (${\triangle }{E_{{{{\rm{H}}}_{2}}{{{\rm{O}}}}}}$) was calculated by the following equation:

$${\triangle }{E_{{{{\rm{H}}}_{2}}{{{\rm{O}}}}}}={E_{{{{\rm{H}}}_{2}}{{{\rm{O}}}}}}+{E_{{{\rm{sub}}}}}-{{E}_{{{\rm{total}}}}}$$

whereas *E*_total_ refers to energy of substrate with corresponding adsorbate, ${\triangle }{E_{{{{\rm{H}}}_{2}}{{{\rm{O}}}}}}$ refer to energy of one H_2_O molecule, *T* is selected by room temperature (298 K), and ZPE refers to zero point energy.

## Supplementary information

Supplementary Information

Peer review file

## Data Availability

All data generated or analyzed during this study are included in this published article and its supplementary information file. Source data are provided with this paper.
