# Supplementary material for: Bottom-up evolution of perovskite clusters into high-activity rhodium nanoparticles toward alkaline hydrogen evolution
Source: Nat Commun. 2023 Jan 17;14:280. doi: 10.1038/s41467-023-35783-y (PMC9845238; doi:10.1038/s41467-023-35783-y)
Supplement: Supplementary file 1 — Supplementary Information [file 41467_2023_35783_MOESM1_ESM.pdf]

SUPPLEMENTARY INFORMATION

Bottom-up Evolution of Perovskite Clusters into High-activity Rhodium Nanoparticles toward Alkaline Hydrogen Evolution

Gaoxin Lin,[ab#] Zhuang Zhang,[ab#] Qiangjian Ju,[ab#] Tong Wu,[ab] Carlo U. Segre,[c] Wei Chen,[d] Hongru Peng,[e] Hui Zhang,[f] Qiunan Liu,[g] Zhi Liu,[ef] Yifan Zhang,[ab] Shuyi Kong,[ab] Yuanlv Mao,[ab] Wei Zhao,[ab] Kazu Suenaga,[g] Fuqiang Huang,[ah]* Jiacheng Wang[abij]*

[a]State Key Lab of High Performance Ceramics and Superfine microstructure, Shanghai Institute of Ceramics, Chinese Academy of Sciences, Shanghai 201899, China

[b]Center of Materials Science and Optoelectronics Engineering, University of Chinese Academy of Sciences, Beijing 100049, China

[c]Department of Physics & Center for Synchrotron Radiation Research and Instrumentation, Illinois Institute of Technology, Chicago, IL 60616, USA

[d]Department of Mechanical, Materials and Aerospace Engineering, Illinois Institute of Technology, Chicago, IL 60616, USA

[e]School of Physical Science and Technology, ShanghaiTech University, Shanghai 201210, China

[f]State Key Laboratory of Functional Materials for Informatics, Shanghai Institute of Microsystem and Information Technology, Chinese Academy of Sciences, Shanghai 200050, China

[g]SANKEN, Osaka University, Ibaraki, 567-0047, Japan

[h]State Key Laboratory of Rare Earth Materials Chemistry and Applications, College of Chemistry and Molecular Engineering, Peking University, Beijing 100871, China

[i]Hebei Provincial Key Laboratory of Inorganic Nonmetallic Materials, College of Materials Science and Engineering, North China University of Science and Technology, Tangshan 063210, China

[j]School of Materials Science and Engineering, Taizhou University, Taizhou, Zhejiang 318000, China.

[#] These authors contribute equally to this work.

Corresponding author: huangfq@mail.sic.ac.cn; jiacheng.wang@mail.sic.ac.cn

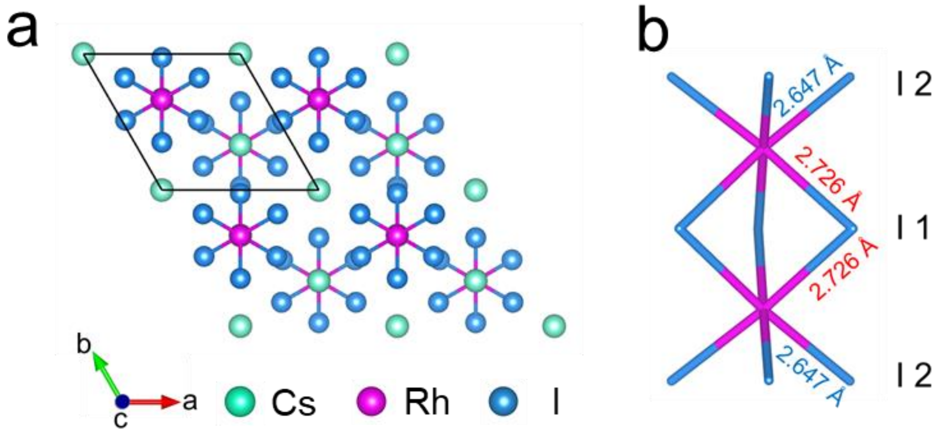

Supplementary Fig. 1 **a** Atomic structure of $Cs_3Rh_2I_9$. **b** The detail of $[Rh_2I_9]^{3-}$ bi-octahedra.

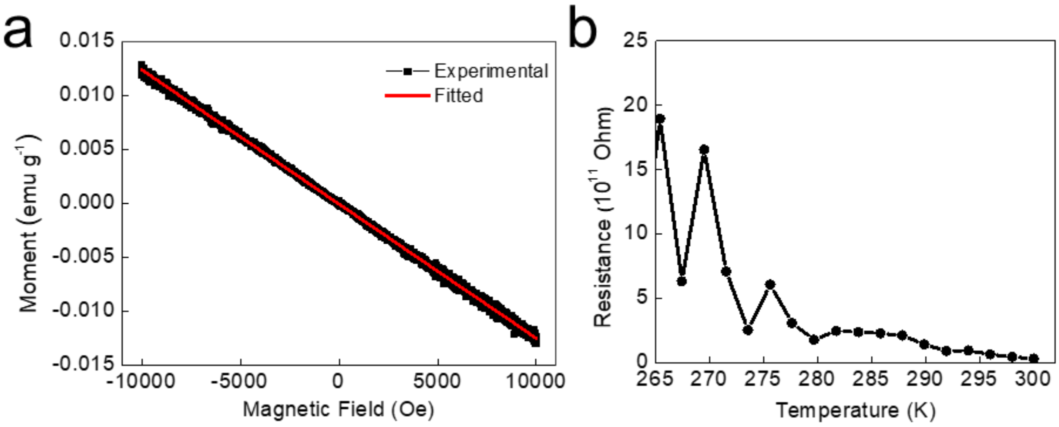

Supplementary Fig. 2 The curves of **a** moment-magnetic field at 3 K and **b** resistance-temperature of $Cs_3Rh_2I_9$ single crystal.

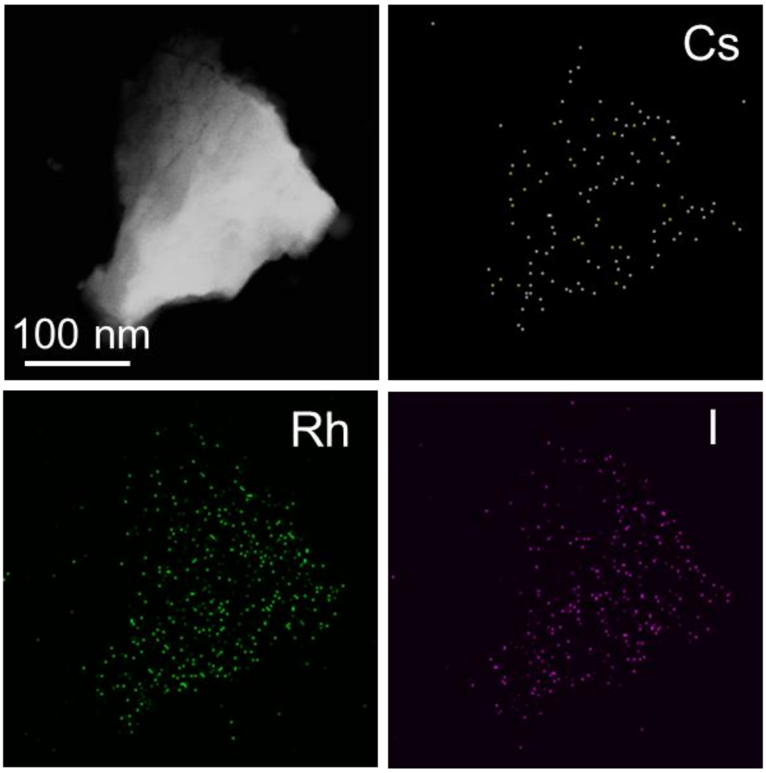

Supplementary Fig. 3 TEM-EDX mapping images of $Cs_3Rh_2I_9$.

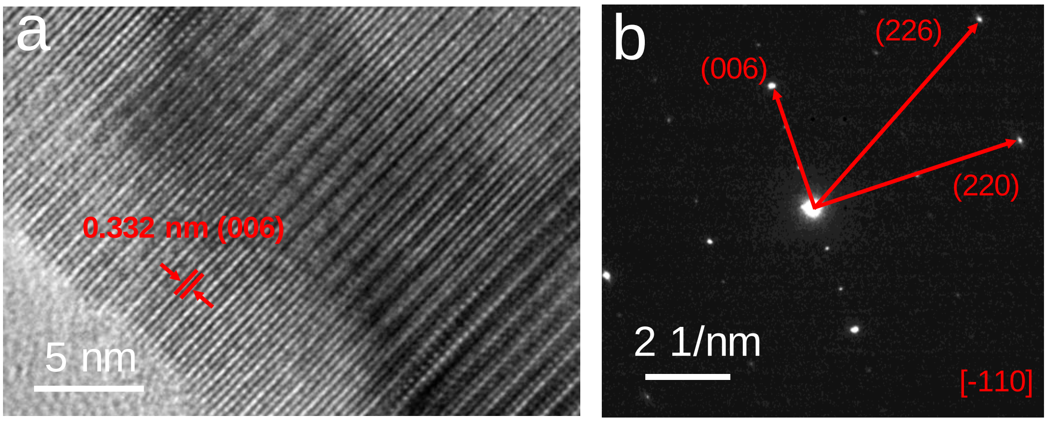

Supplementary Fig. 4 a HRTEM of $Cs_3Rh_2I_9$. **b** The corresponding selected area electron diffraction image.

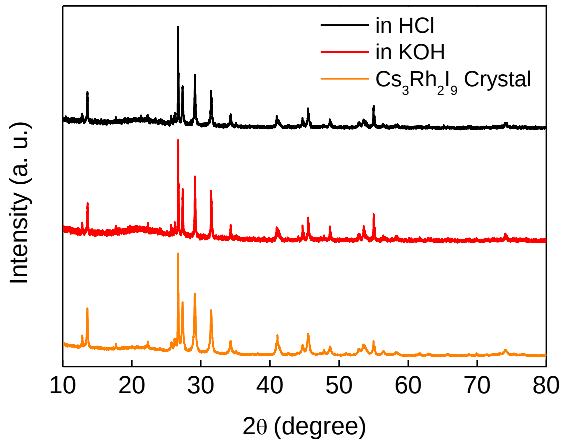

Supplementary Fig. 5 The XRD patterns of $Cs_3Rh_2I_9$ after immersion in 1.0 M HCl or 1.0 M KOH for 7 days.

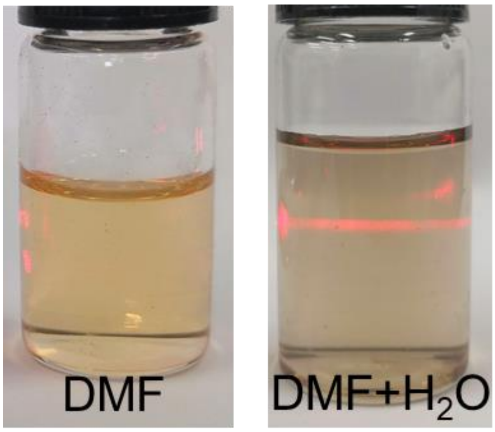

Supplementary Fig. 6 The photographs of $Cs_3Rh_2I_9$ in DMF without Tyndall effect (left) and $Cs_3Rh_2I_9$ in H_2O/DMF with Tyndall effect (right).

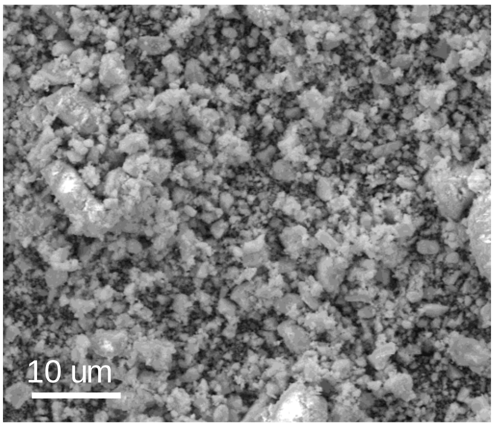

Supplementary Fig. 7 SEM image of precipitated $Cs_3Rh_2I_9$.

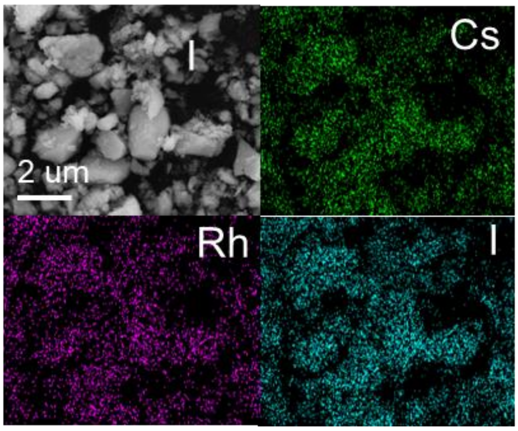

Supplementary Fig. 8 SEM-EDX mapping images of precipitated $Cs_3Rh_2I_9$.

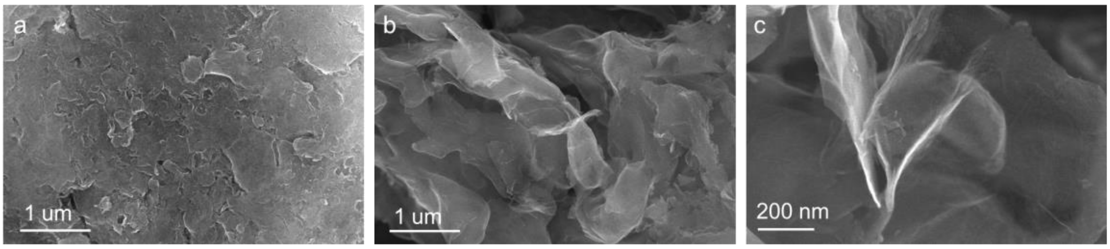

Supplementary Fig. 9 SEM images of $Cs_3Rh_2I_9$/NC.

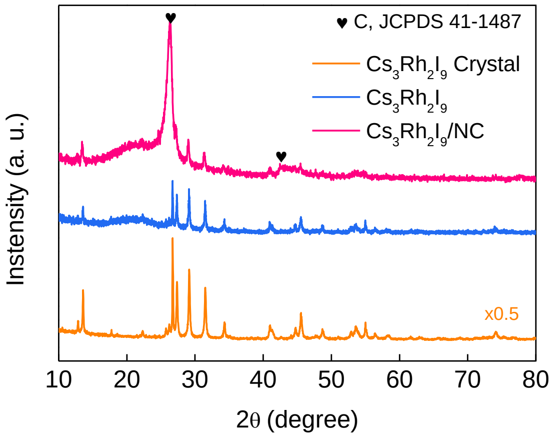

Supplementary Fig. 10 XRD patterns of $Cs_3Rh_2I_9$ crystal, $Cs_3Rh_2I_9$ and $Cs_3Rh_2I_9$/NC.

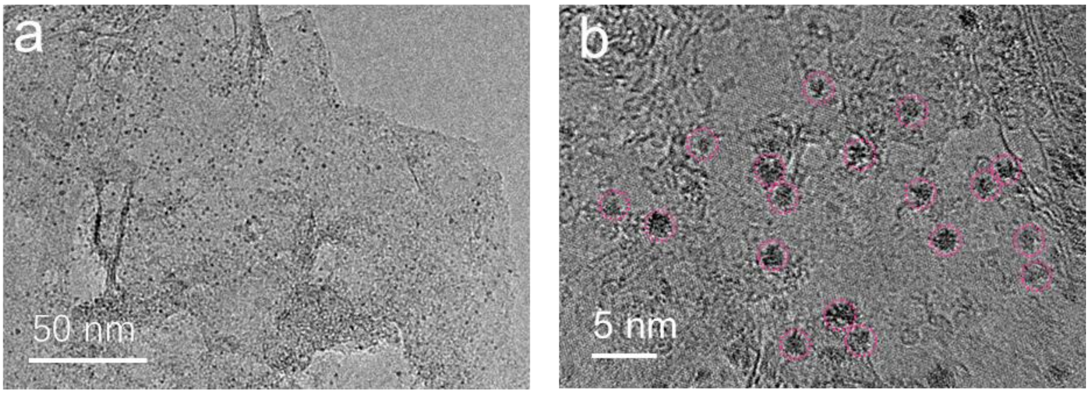

Supplementary Fig. 11 a TEM image of $Cs_3Rh_2I_9$/NC. **b** HRTEM image with the red circles showing the $Cs_3Rh_2I_9$ clusters.

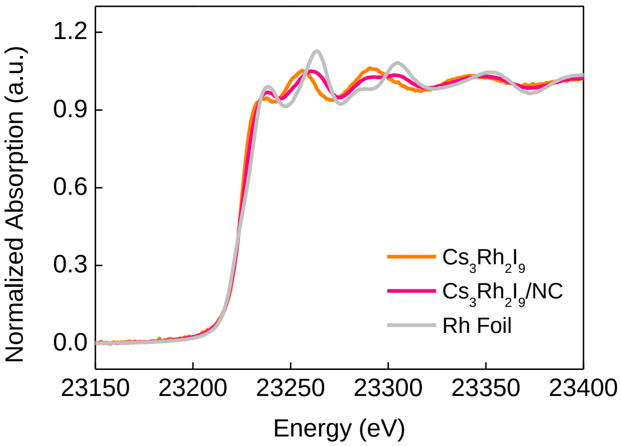

Supplementary Fig. 12 The X-ray absorption near edge structure spectra of Rh K-edge for $Cs_3Rh_2I_9$, $Cs_3Rh_2I_9$/NC, and Rh foil.

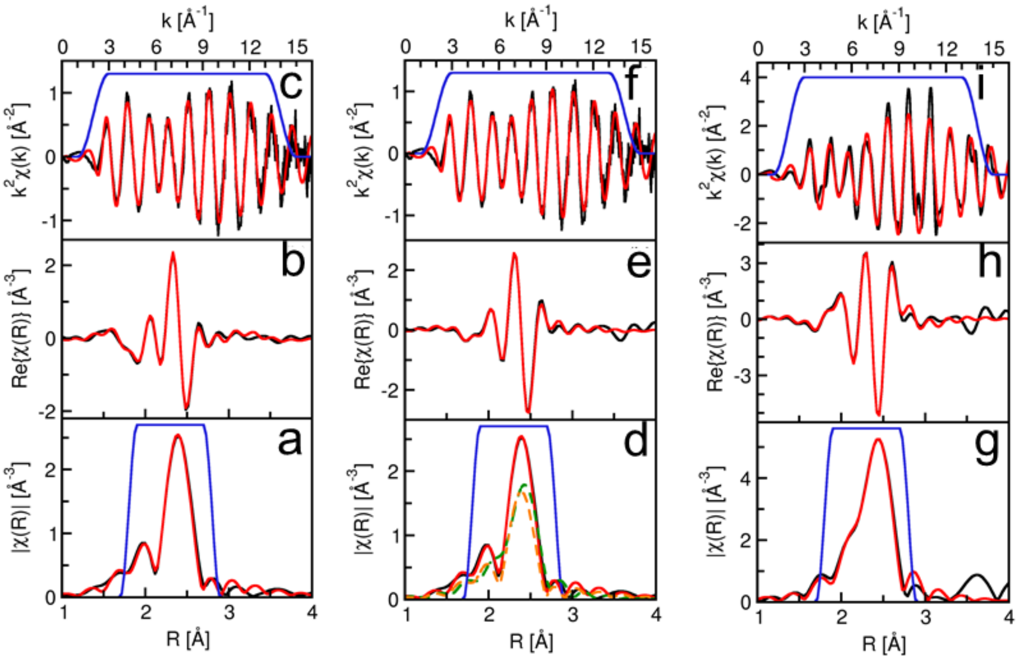

Supplementary Fig. 13 EXAFS data and fitting results. **a-c** Rh edge of $Cs_3Rh_2I_9$ sample showing magnitude of FT, real part of FT and weighted χ(k). **d-f** Rh edge of $Cs_3Rh_2I_9$/NC sample showing magnitude of FT, real part of FT and weighted χ(k). **g-i** Rh edge of Rh foil sample showing magnitude of FT, real part of FT and weighted χ(k). In all figures, the data is shown in black, the fit in red and the FT and fitting windows in blue.

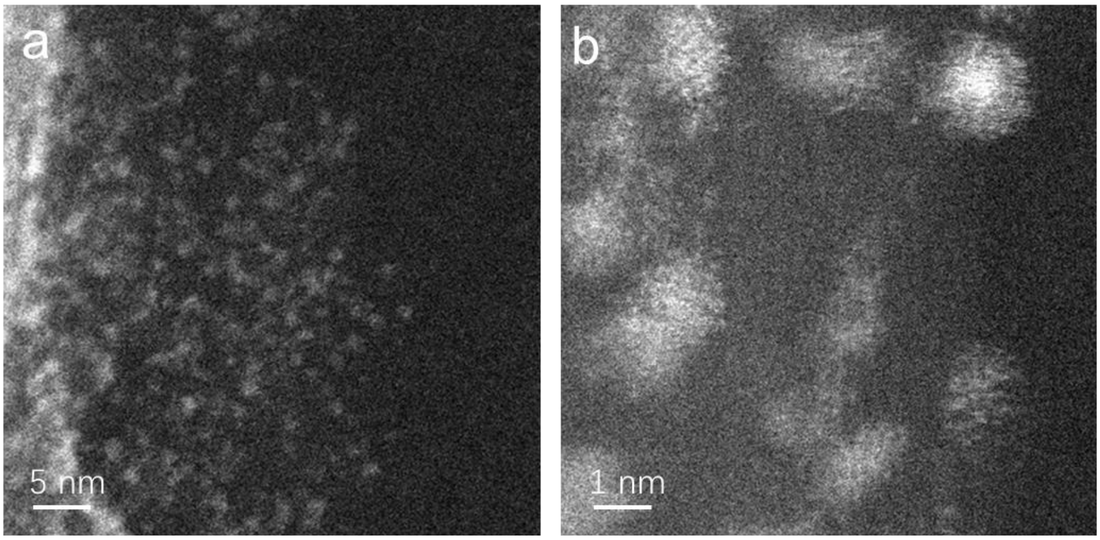

Supplementary Fig. 14 a HAADF-STEM of $Cs_3Rh_2I_9$/NC and **b** decomposed $Cs_3Rh_2I_9$/NC. The $Cs_3Rh_2I_9$ nano-clusters are unstable under the high-energy measurement.

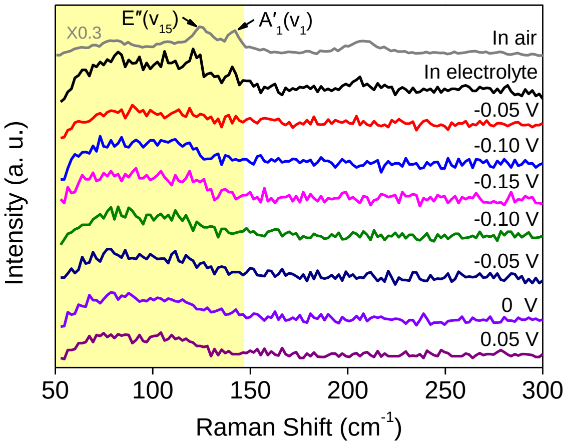

Supplementary Fig. 15 The in-situ Raman spectra of $Cs_3Rh_2I_9$/NC under the CV measurement.

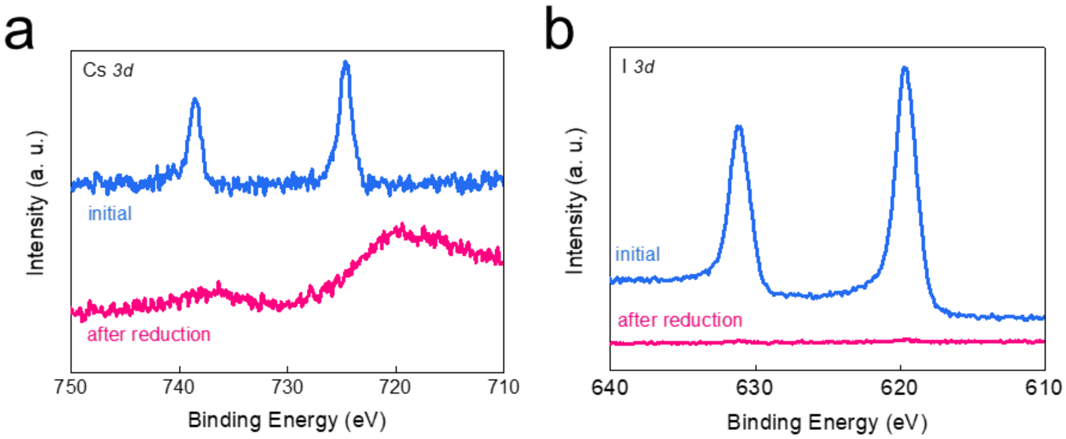

Supplementary Fig. 16 High resolution XPS spectra of **a)** Cs *3d* and **b)** I *3d* for $Cs_3Rh_2I_9$/NC before and after reduction.

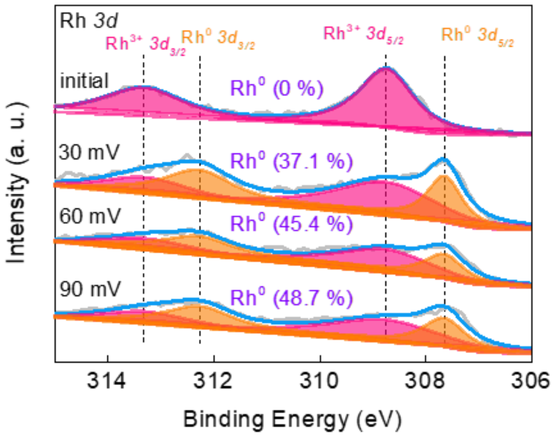

Supplementary Fig. 17 The ex-situ XPS spectra for $Cs_3Rh_2I_9$/NC of different overpotential under the CV measurement.

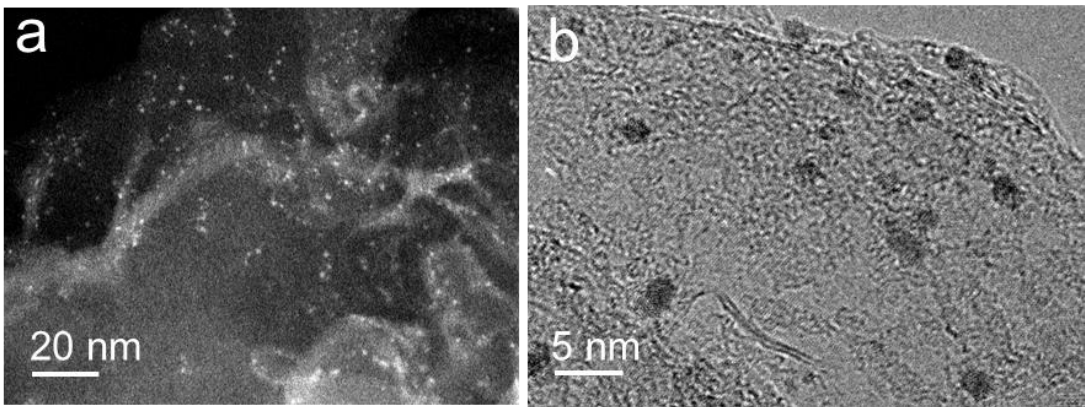

Supplementary Fig. 18 a HAADF image and **b** HRTEM of $Cs_3Rh_2I_9$/NC after reduction.

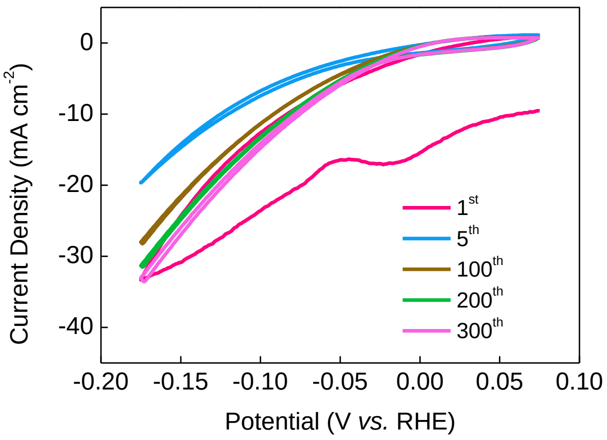

Supplementary Fig. 19 The CV curves from 1st to 300th cycle at 100 mV s^{-1} for $Cs_3Rh_2I_9$.

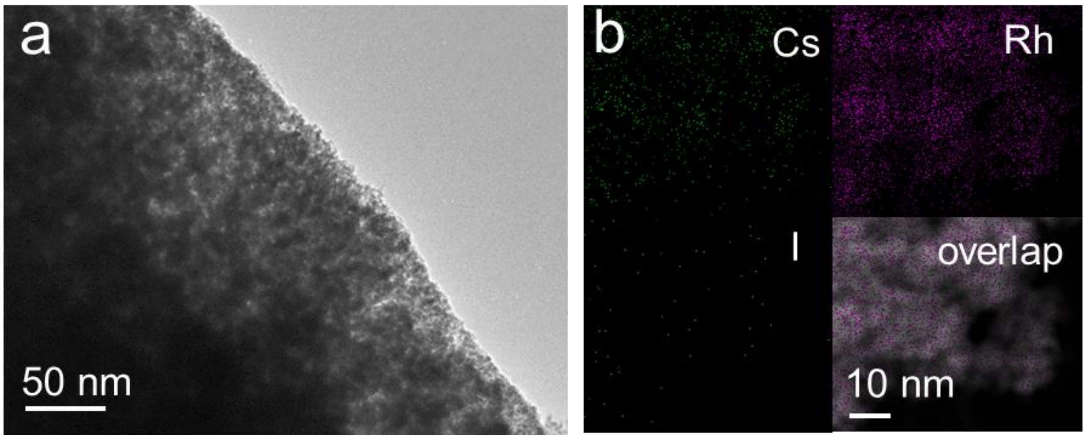

Supplementary Fig. 20 a TEM, **b** EDX images of $Cs_3Rh_2I_9$ after reduction ($Cs_3Rh_2I_9$-R).

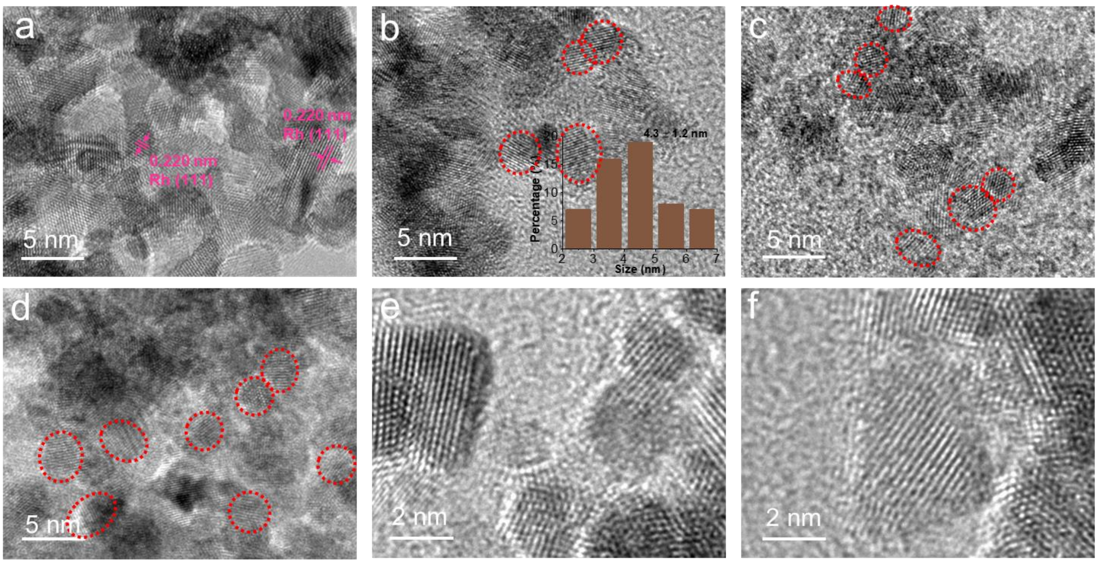

Supplementary Fig. 21 HRTEM of $Cs_3Rh_2I_9$-R. The inset in **b** shows particle size distribution.

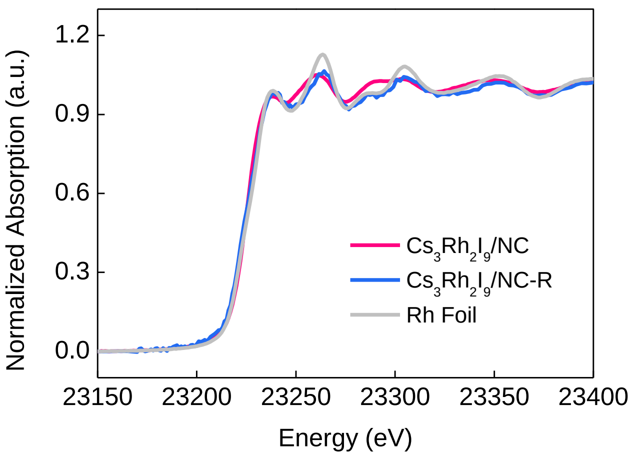

Supplementary Fig. 22 The X-ray absorption near edge structure spectra of $Cs_3Rh_2I_9$/NC, $Cs_3Rh_2I_9$/NC-R, and Rh foil at Rh K-edge.

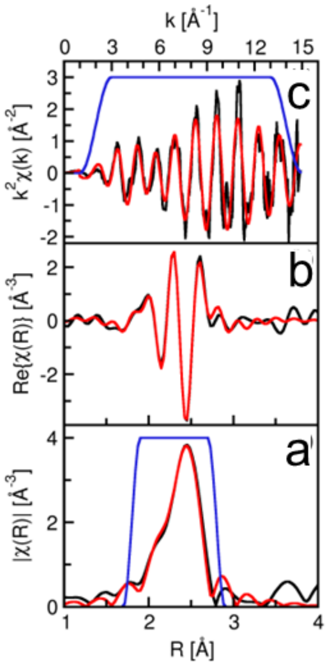

Supplementary Fig. 23 EXAFS data and fitting results. **a-c** Rh edge of reduced $Cs_3Rh_2I_9$/NC sample showing magnitude of FT, real part of FT and weighted χ(k). In all figures, the data is shown in black, the fit is shown in red, and the FT and fitting windows are shown in blue.

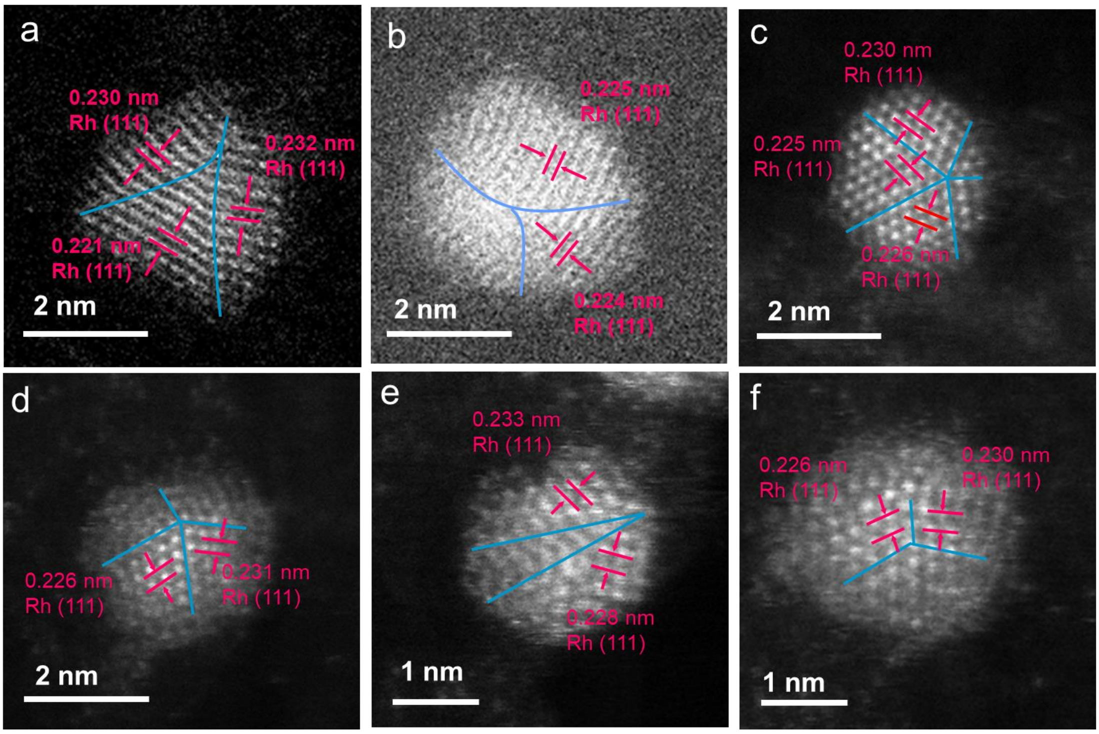

Supplementary Fig. 24 HAADF-STEM images of reduced $Cs_3Rh_2I_9$/NC ($Cs_3Rh_2I_9$/NC-R). And these images show that the $Cs_3Rh_2I_9$/NC-R is composed of twinned Rh nanoparticles.

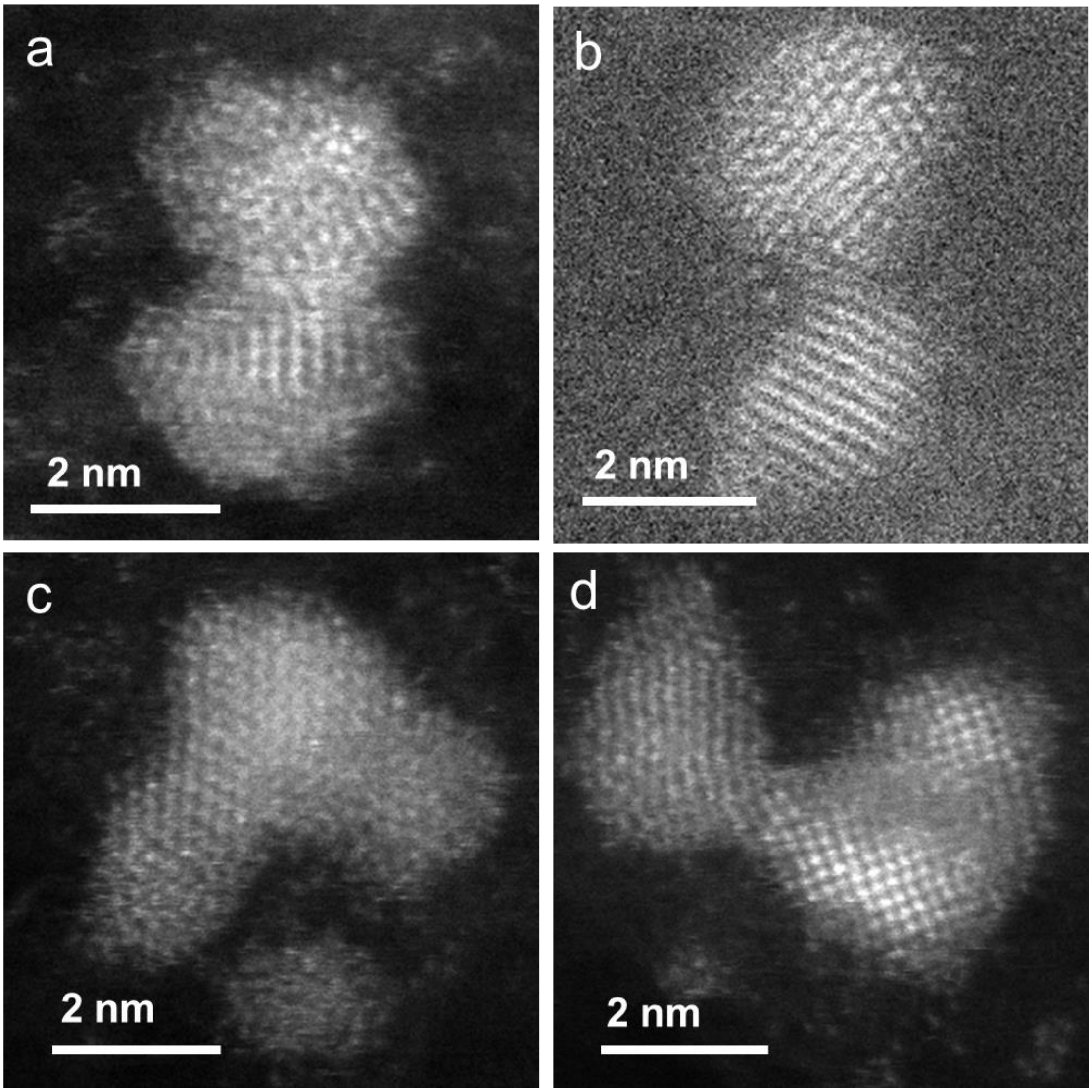

Supplementary Fig. 25 HAADF-STEM images of adjacent Rh particles in reduced $Cs_3Rh_2I_9$/NC ($Cs_3Rh_2I_9$/NC-R).

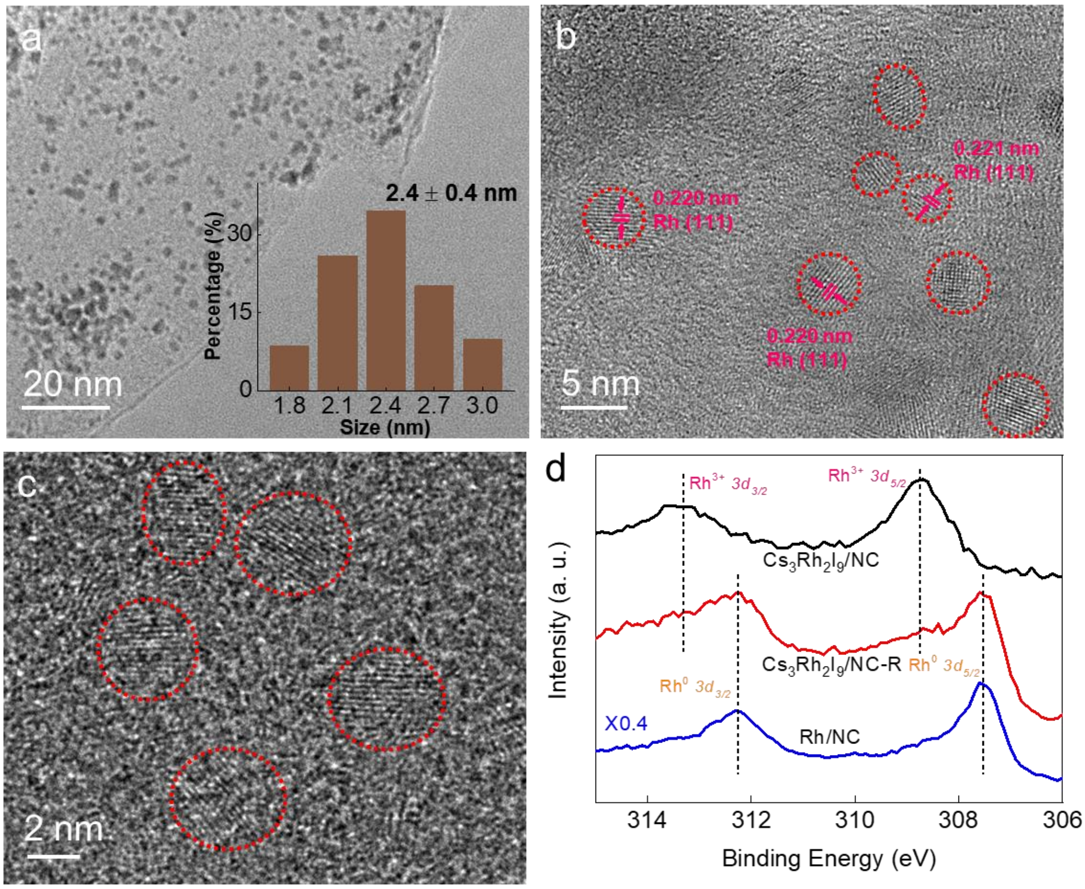

Supplementary Fig. 26 a TEM images of Rh/NC. The inset shows the Rh particle size distribution. **b** and **c** HRTEM of Rh/NC. The red circles show the Rh particle. **d** XPS spectra of Rh *3d* for $Cs_3Rh_2I_9$/NC, $Cs_3Rh_2I_9$/NC-R and Rh/NC.

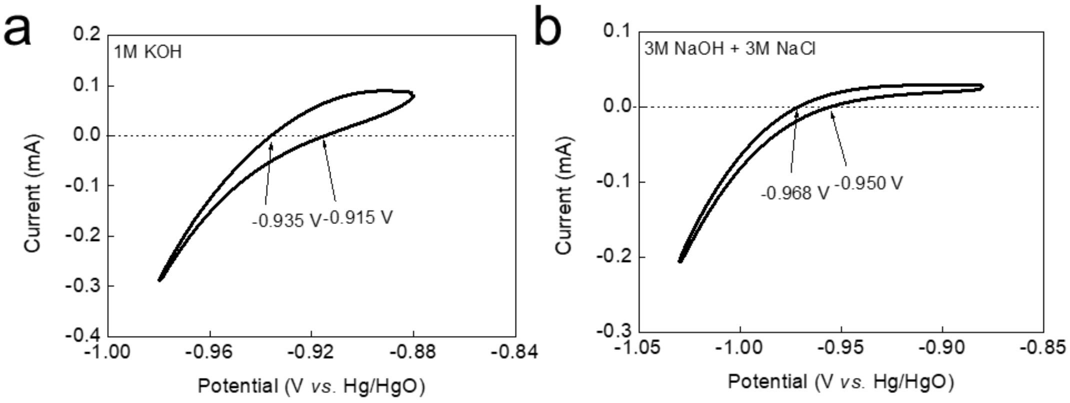

Supplementary Fig. 27 CV curves of RHE calibration in 1.0 M KOH **(a)** and 3.0 M NaOH+3.0 M NaCl **(b)**.

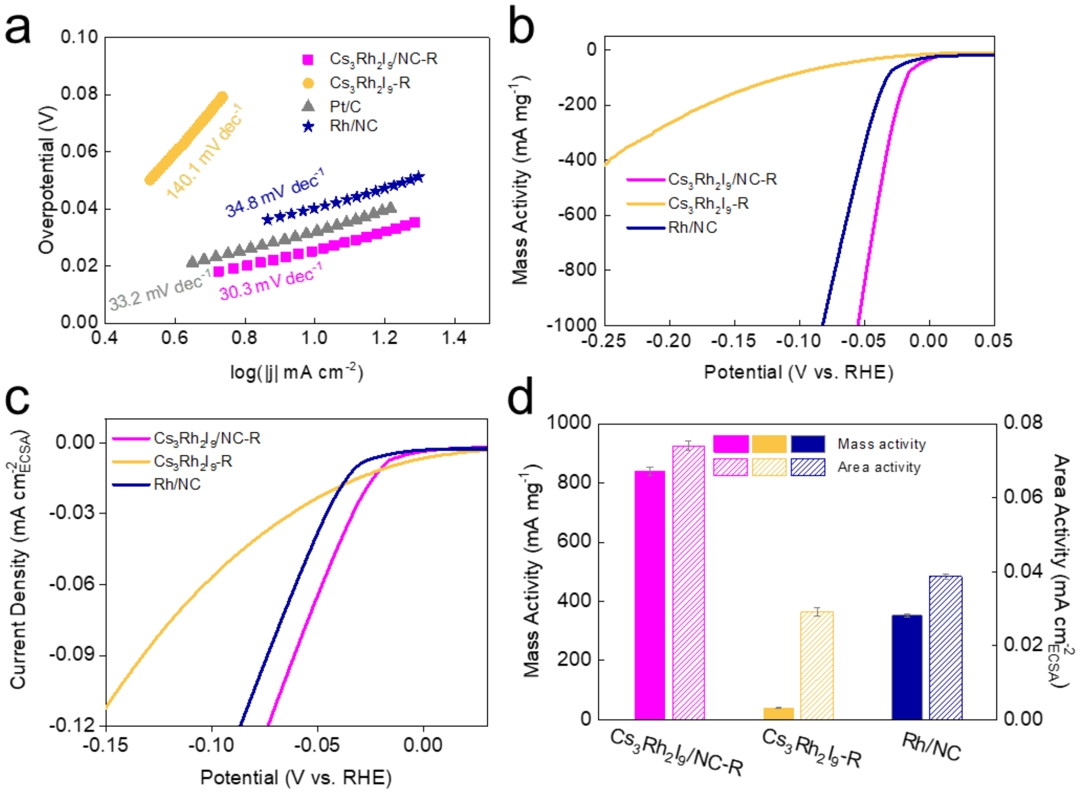

Supplementary Fig. 28 The HER activity of different electrocatalysts on rotating GCE (1600 rpm) in 1.0 M KOH. **a** Tafel plots corresponding to the curves in Figure 5a. **b** Mass activity normalized to the mass of Rh. **c** Area activity normalized to the ECSA. **d** Comparison of mass activity and area activity at the overpotential of 50 mV. The catalyst loading amount on GCE is 0.764 mg cm^{-2}. All data shows the mean and standard deviation through three repeated measurements. For Rh-based samples, the calculated Rh loading amounts on GCE are 0.045, 0.090 and 0.053 mg cm^{-2} for $Cs_3Rh_2I_9$/NC-R, $Cs_3Rh_2I_9$-R, and Rh/NC, respectively.

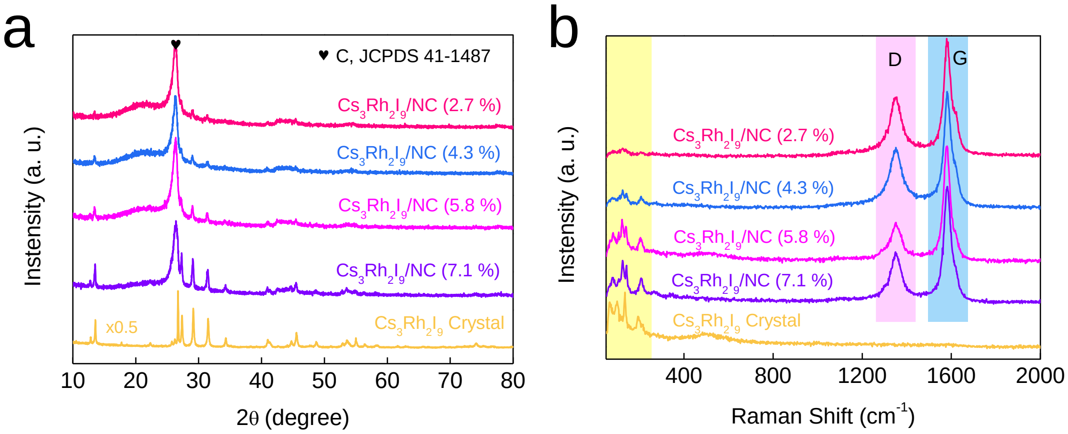

Supplementary Fig. 29 a XRD patterns and **b** Raman spectra of $Cs_3Rh_2I_9$/NC with different Rh contents.

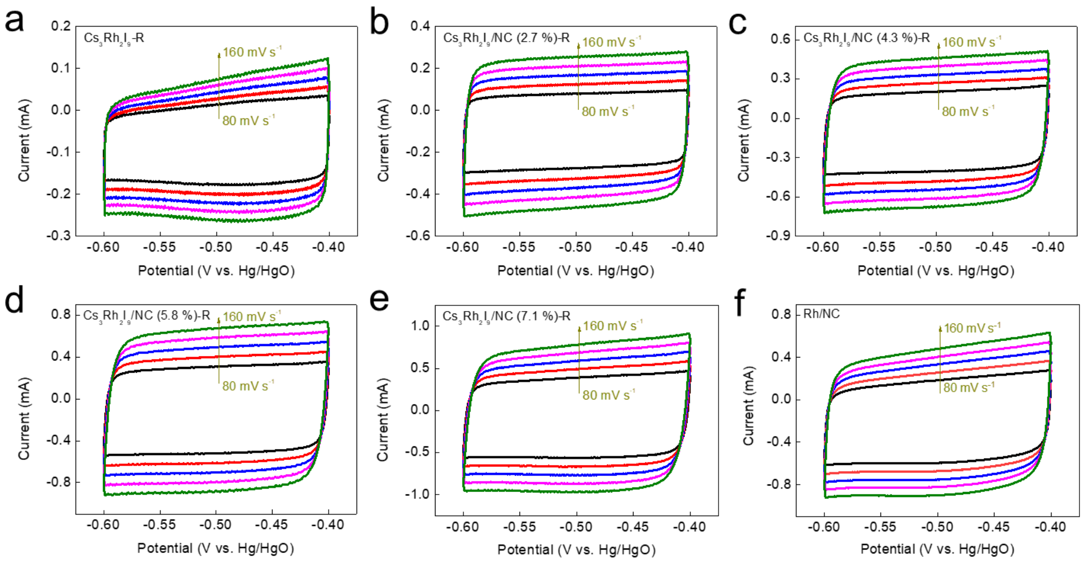

Supplementary Fig. 30 CV curves of $Cs_3Rh_2I_9$-R **(a)**, $Cs_3Rh_2I_9$/NC (2.7 wt.%)-R **(b)**, $Cs_3Rh_2I_9$/NC (4.3 wt.%)-R **(c)**, $Cs_3Rh_2I_9$/NC (5.8 wt.%)-R **(d)**, $Cs_3Rh_2I_9$/NC (7.1 wt.%)-R **(e)**, and Rh/NC **(f)** at different scan rates in 1.0 M KOH.

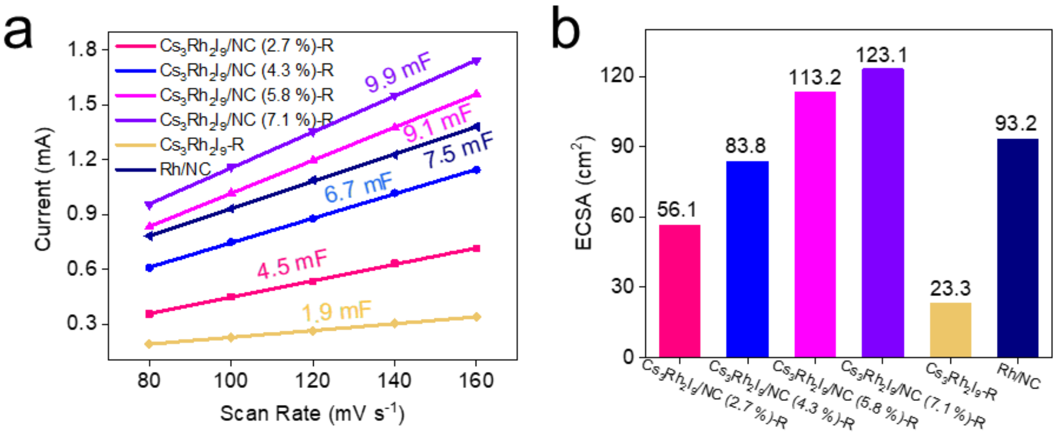

Supplementary Fig. 31 **a** The currents (-0.50 V *vs*. Hg/HgO) as a function of scan rate, and **b** the electrochemical surface area (ECSA) for various Rh-based samples.

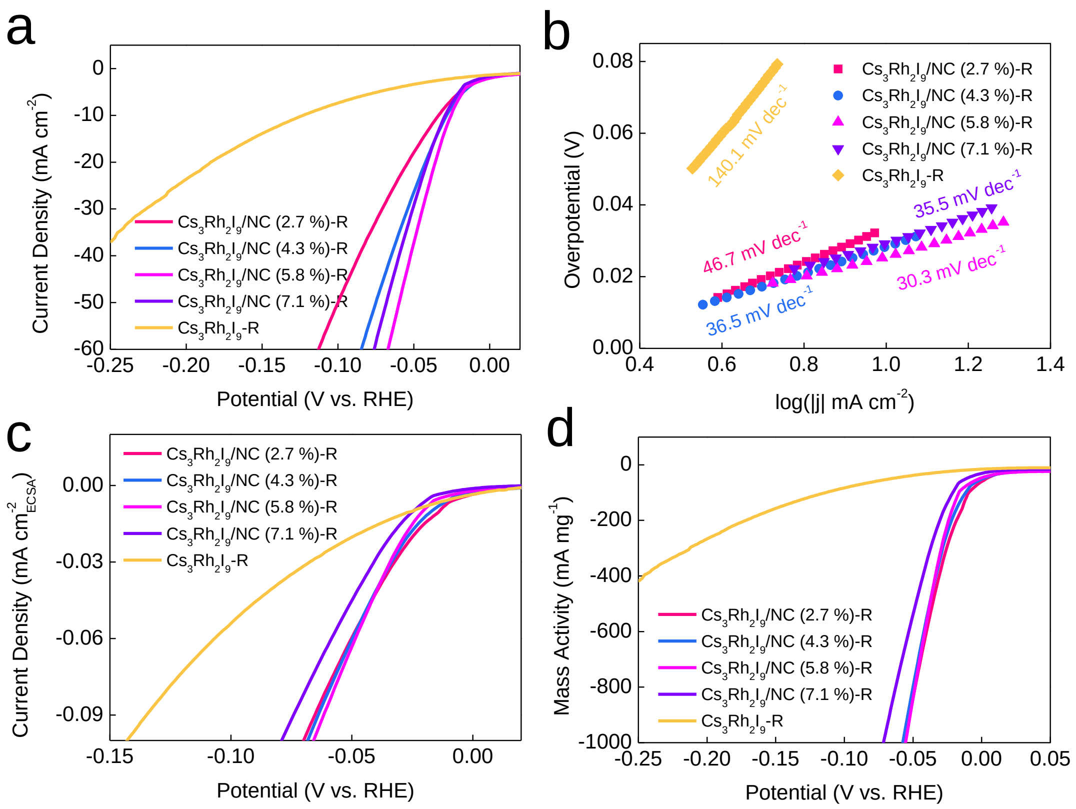

Supplementary Fig. 32 The HER activity of different Rh-based samples coated on rotating GCE (1600 rpm) in 1.0 M KOH. **a** LSV curves. **b** Tafel plots. **c** The area activity normalized to the ECSA. **d** Mass activity normalized to the mass of Rh. The catalyst loading amount on GCE is 0.764 mg cm^{-2}.

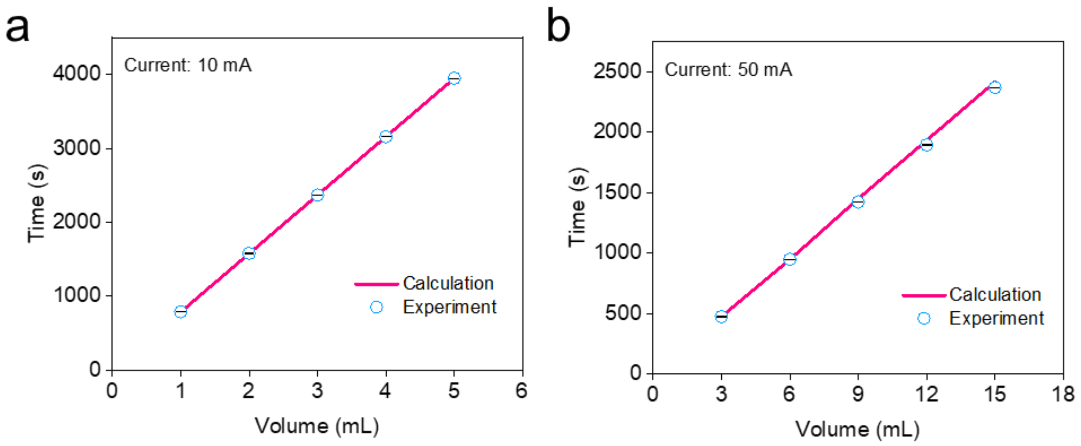

Supplementary Fig. 33 Total amount of H_2 produced over time at a current of 10 mA (**a**) and 50 mA (**b**) in 1 M KOH for $Cs_3Rh_2I_9$/NC-R, showing the nearly 100% faradaic efficiency.

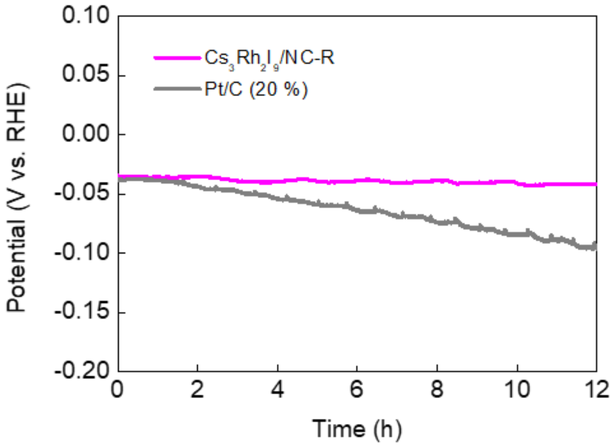

Supplementary Fig. 34 Long-term HER stability at the current density of 10 mA cm^{-2} in 1.0 M KOH.

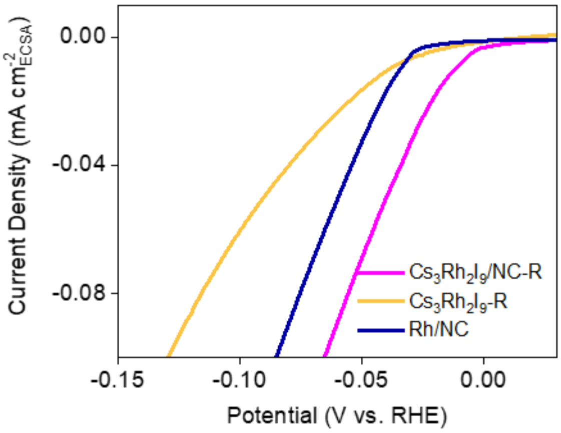

Supplementary Fig. 35 Area activity normalized to ECSA in chlorine-alkali electrolyte for $Cs_3Rh_2I_9$/NC-R, $Cs_3Rh_2I_9$-R, and Rh/NC.

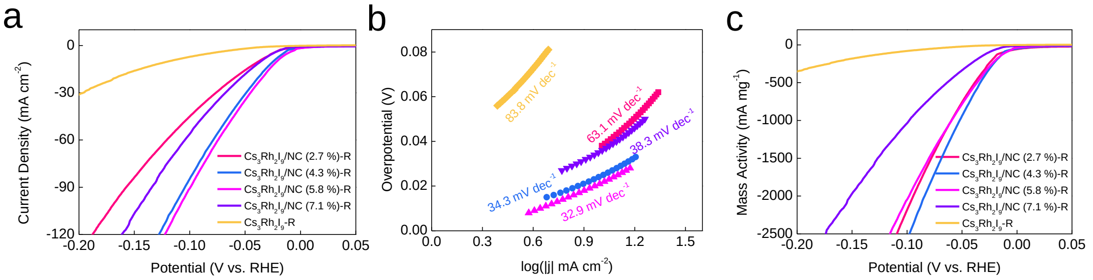

Supplementary Fig. 36 The HER activity of different Rh-based samples coated on rotating GCE (1600 rpm) in chlorine-alkali electrolyte. **a** LSV curves. **b** Tafel plots. **c** Mass activity normalized to the mass of Rh. The catalyst loading amount on GCE is 0.764 mg cm^{-2}.

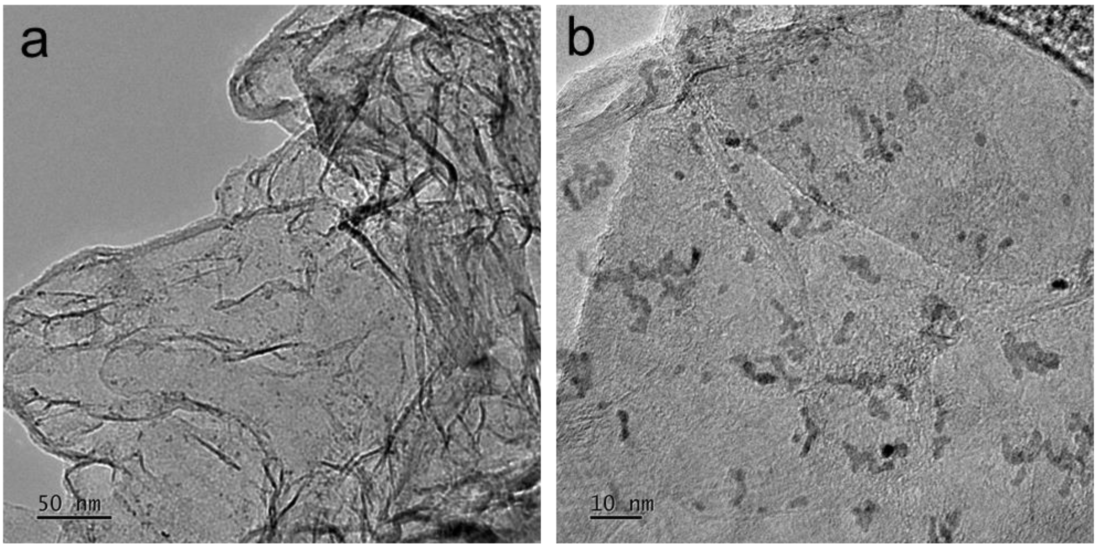

Supplementary Fig. 37 a TEM and **b** HRTEM images of $Cs_3Rh_2I_9$/NC-R after the durable measurement.

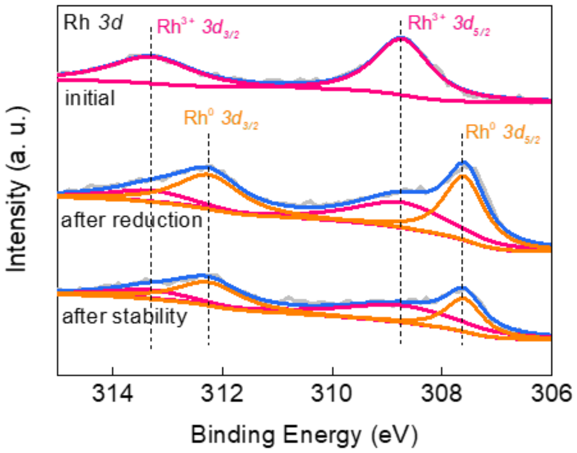

Supplementary Fig. 38 XPS spectra of $Cs_3Rh_2I_9$/NC-R after the durable measurement.

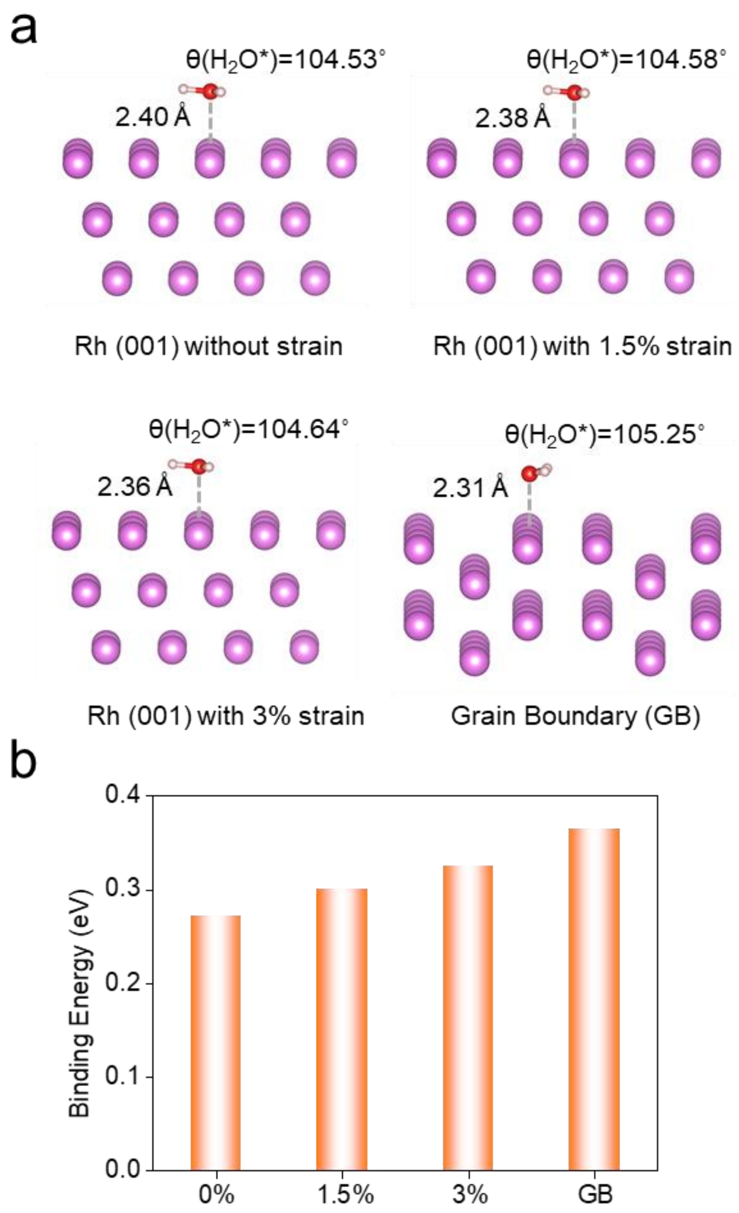

Supplementary Fig. 39 a Structural models of adsorbed water molecule on Rh (001), Rh (001) with tensions, and Rh with (110) GB. **b** Binding energy of water molecule.

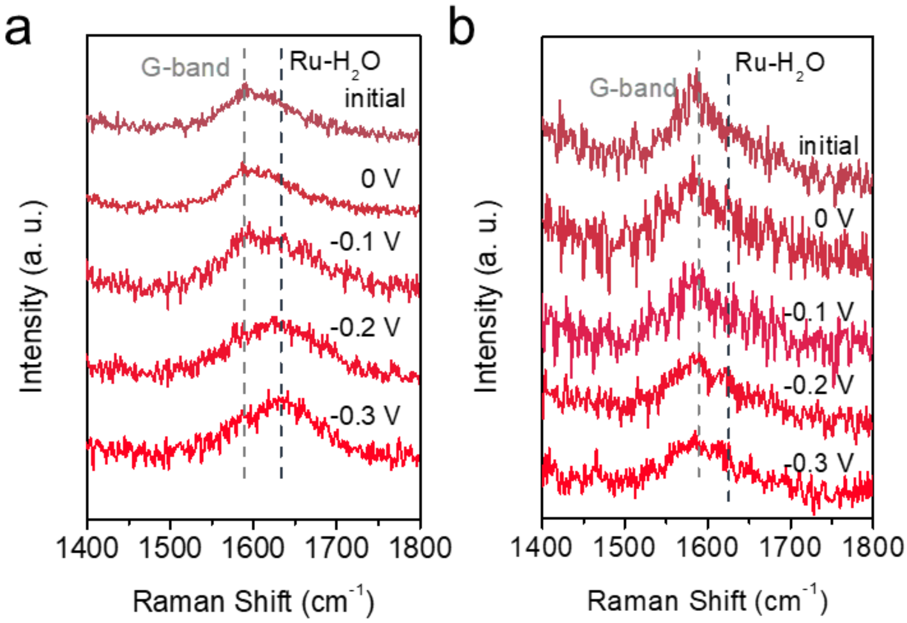

Supplementary Fig. 40 In situ Raman spectra of interfacial water on $Cs_3Rh_2I_9$/NC-R (**a**) and Rh/NC (**b**).

Supplementary Table 1. Crystallographic data and structural refinement for $Cs_3Rh_2I_9$.

Chemical formula	$Cs_3Rh_2I_9$
M_r	1746.67
Crystal system, space group	Hexagonal, $P6_3/mmc$
Temperature (K)	298
a, c (Å)	7.9644 (7), 20.0225 (18)
V (Å^3)	1099.9 (2)
Z	2
Radiation type	Mo $K\alpha$
μ (mm^{-1})	19.00
No. of measured, independent and observed [$I > 2\sigma(I)$] reflections	5410, 403, 322
R_{int}	0.035
$(\sin \theta/\lambda)_{max}$ (Å^{-1})	0.595
R [$F^2 > 2\sigma(F^2)$], wR (F^2), S	0.054, 0.140, 1.15
No. of reflections	403
No. of parameters	19
$\Delta\rangle_{max}$, $\Delta\rangle_{min}$ (e Å^{-3})	1.52, -1.33

Supplementary Table 2. Fractional atomic coordinates, equivalent isotropic displacement parameters (Å^2) of $Cs_3Rh_2I_9$.

Atom	Wyck	x	y	z	U_{iso}*/U_{eq}
Cs 1	2*b*	0	0	1/4	0.0698 (12)
Cs 2	4*f*	2/3	1/3	0.57460 (15)	0.0739 (10)
Rh	4*f*	2/3	1/3	0.33522 (13)	0.0547 (10)
I 1	6*h*	0.51255 (16)	0.48745 (16)	1/4	0.0592 (9)
I 2	12*k*	0.82483 (13)	0.6497 (3)	0.41004 (7)	0.0660 (8)

Supplementary Table 3. The molar ratio of Rh, I and Cs elements from Figure S3.

Element	Line type	K factor	Absorption correction	wt%	wt% sigma	Atomic percentage
Rh	L	1.848	1.00	11.76	0.48	14.25
I	L	1.986	1.00	66.52	0.66	65.36
Cs	L	2.020	1.00	21.73	0.61	20.39
total				100.00		100.00

Supplementary Table 4. Structure parameters extracted from the Rh K-edge EXAFS fittings of Rh foil, $Cs_3Rh_2I_9$, and $Cs_3Rh_2I_9$/NC.

<table>
<tr><td>Samples</td><td>Path</td><td colspan="2">N</td><td>σ^2 (Å^2)</td><td>ΔE (eV)</td><td>R (Å)</td></tr>
<tr><td>Rh foil</td><td>Rh–Rh</td><td colspan="2">12</td><td>0.0035±0.0005</td><td>3.0±0.7</td><td>2.682±0.004</td></tr>
<tr><td>$Cs_3Rh_2I_9$</td><td>Rh–I</td><td colspan="2">5.0±0.3</td><td>0.0041±0.0004</td><td>0.3±0.4</td><td>2.651±0.003</td></tr>
<tr><td colspan="7"></td></tr>
<tr><td></td><td>Path</td><td colspan="2">Fraction</td><td>σ^2 (Å^2)</td><td>ΔE (eV)</td><td>R (Å)</td></tr>
<tr><td rowspan="2">$Cs_3Rh_2I_9$/NC*</td><td>Rh–I</td><td>0.43</td><td rowspan="2">±0.13</td><td>0.004±0.001</td><td rowspan="2">0.5±1.4</td><td>2.651</td></tr>
<tr><td>Rh–Rh</td><td>0.57</td><td>0.004±0.001</td><td>2.682</td></tr>
</table>

*The fitting model used for $Cs_3Rh_2I_9$/NC takes the Rh-Rh path from Rh foil and the Rh-I path from $Cs_3Rh_2I_9$ fits and holds their distance constant, allowing path and the relative fraction of the paths and the σ^2 for each path to vary.

Supplementary Table 5. ICP-MS results of Rh/Cs/I content in $Cs_3Rh_2I_9$/NC-R.

Elements	Mass ratio	Atomic ratio
Rh:Cs:I	1:0.0084:0.0024	1:0.0065:0.0019

Supplementary Table 6. Structure parameters extracted from the Rh K-edge EXAFS fittings of $Cs_3Rh_2I_9$/NC-R.

Samples	Path	N	σ^2 (Å^2)	ΔE (eV)	R (Å)
$Cs_3Rh_2I_9$/NC-R	Rh–Rh	8.0±1.0	0.0031±0.0006	3.8±0.8	2.683±0.005

Supplementary Table 7. HER activity of recent advanced electrocatalysts in 1 M KOH.

Catalysts	Overpotential at 10 mA cm^{-2}	Tafel slope	Reference
PS-MoNi@NF	30 mV	37 mV dec^{-1}	[1]
Ni-Mo-N/CFC	43 mV	70 mV dec^{-1}	[2]
Ni@C	27 mV	38 mV dec^{-1}	[3]
Ru_1/D-NiFe LDH	18 mV	29 mV dec^{-1}	[4]
Mo-NiO/Ni	50 mV	86 mV dec^{-1}	[5]
F-Ni_3S_4/NF	29 mV	46 mV dec^{-1}	[6]
Ru@Ni-MOF	22 mV	40 mV dec^{-1}	[7]
Pt–Ni NTA	23 mV	38 mV dec^{-1}	[8]
MoO_2-FeP@C	103 mV	48 mV dec^{-1}	[9]
Cu–Ni nanocages	140 mV	79 mV dec^{-1}	[10]
Ni-CeF_3-VN	33 mV	37 mV dec^{-1}	[11]
Pt_{SA}-NiO/Ni	26 mV	27 mV dec^{-1}	[12]
Cr–Ni NHs	75 mV	72 mV dec^{-1}	[13]
Co_1/PCN	89 mV	52 mV dec^{-1}	[14]
Ni_5P_4-Ru	54 mV	52 mV dec^{-1}	[15]
FD-MoS_2	164 mV	36 mV dec^{-1}	[16]
Pt_1/N-C	46 mV	37 mV dec^{-1}	[17]
$NiRu_{0.13}$-BDC	34 mV	32 mV dec^{-1}	[18]
$Cs_3Rh_2I_9$/NC-R	25 mV	30 mV dec^{-1}	This work

References

1. Song J*, et al.* Phase-Separated Mo–Ni Alloy for Hydrogen Oxidation and Evolution Reactions with High Activity and Enhanced Stability. *Adv. Energy Mater.* **11**, 2003511 (2021).
2. Li Y, Wei X, Chen L, Shi J, He M. Nickel-molybdenum nitride nanoplate electrocatalysts for concurrent electrolytic hydrogen and formate productions. *Nat. Commun.* **10**, 5335 (2019).
3. Gu Y, Xi B, Wei R, Fu Q, Qain Y, Xiong S. Sponge Assembled by Graphene Nanocages with Double Active Sites to Accelerate Alkaline HER Kinetics. *Nano Lett.* **20**, 8375-8383 (2020).
4. Zhai P*, et al.* Engineering single-atomic ruthenium catalytic sites on defective nickel-iron layered double hydroxide for overall water splitting. *Nat. Commun.* **12**, 4587 (2021).
5. Huang J*, et al.* Boosting Hydrogen Transfer during Volmer Reaction at Oxides/Metal Nanocomposites for Efficient Alkaline Hydrogen Evolution. *ACS Energy Lett.* **4**, 3002-3010 (2019).
6. Wang J*, et al.* Water Dissociation Kinetic-Oriented Design of Nickel Sulfides via Tailored Dual Sites for Efficient Alkaline Hydrogen Evolution. *Adv. Funct. Mater.* **31**, 2008578 (2021).
7. Deng L*, et al.* Electronic modulation caused by interfacial Ni-O-M (M= Ru, Ir, Pd) bonding for accelerating hydrogen evolution kinetics. *Angew. Chem. Int. Ed.* **133**, 22450-22456 (2021).
8. Nairan A*, et al.* Proton selective adsorption on Pt–Ni nano-thorn array electrodes for superior hydrogen evolution activity. *Energy Environ. Sci.* **14**, 1594-1601 (2021).
9. Yang G*, et al.* Interfacial Engineering of MoO2-FeP Heterojunction for Highly Efficient Hydrogen Evolution Coupled with Biomass Electrooxidation. *Adv. Mater.* **32**, 2000455 (2020).
10. Li Z*, et al.* Mesoporous Hollow Cu–Ni Alloy Nanocage from Core–Shell Cu@Ni Nanocube for Efficient Hydrogen Evolution Reaction. *ACS Catal.* **9**, 5084-5095 (2019).

11. Zhou P, *et al.* Construction of Nickel-Based Dual Heterointerfaces towards Accelerated Alkaline Hydrogen Evolution via Boosting Multi-Step Elementary Reaction. *Adv. Funct. Mater.* **31**, 2104827 (2021).

12. Zhou KL, *et al.* Platinum single-atom catalyst coupled with transition metal/metal oxide heterostructure for accelerating alkaline hydrogen evolution reaction. *Nat. Commun.* **12**, 3783 (2021).

13. Kim J, *et al.* Tailoring Binding Abilities by Incorporating Oxophilic Transition Metals on 3D Nanostructured Ni Arrays for Accelerated Alkaline Hydrogen Evolution Reaction. *J. Am. Chem. Soc.* **143**, 1399-1408 (2021).

14. Cao L, *et al.* Identification of single-atom active sites in carbon-based cobalt catalysts during electrocatalytic hydrogen evolution. *Nat. Catal.* **2**, 134-141 (2019).

15. He Q, *et al.* Achieving Efficient Alkaline Hydrogen Evolution Reaction over a Ni5P4 Catalyst Incorporating Single-Atomic Ru Sites. *Adv. Mater.* **32**, 1906972 (2020).

16. Xu J, *et al.* Frenkel-defected monolayer MoS2 catalysts for efficient hydrogen evolution. *Nat. Commun.* **13**, 2193 (2022).

17. Fang S, *et al.* Uncovering near-free platinum single-atom dynamics during electrochemical hydrogen evolution reaction. *Nat. Commun.* **11**, 1029 (2020).

18. Sun Y, *et al.* Modulating electronic structure of metal-organic frameworks by introducing atomically dispersed Ru for efficient hydrogen evolution. *Nat. Commun.* **12**, 1369 (2021).
